# Physiological Importance of Molybdate Transporter Family 1 in Feeding the Molybdenum Cofactor Biosynthesis Pathway in *Arabidopsis thaliana*

**DOI:** 10.3390/molecules27103158

**Published:** 2022-05-15

**Authors:** Rieke Minner-Meinen, Jan-Niklas Weber, Sarah Kistner, Paul Meyfarth, Merve Saudhof, Lena van den Hout, Jutta Schulze, Ralf-Rainer Mendel, Robert Hänsch, David Kaufholdt

**Affiliations:** 1 Institut für Pflanzenbiologie, Technische Universität Braunschweig, Humboldtstrasse 1, D-38106 Braunschweig, Germany; riemeine@tu-braunschweig.de (R.M.-M.); jan-niklas.weber@tu-braunschweig.de (J.-N.W.); s.kistner@tu-braunschweig.de (S.K.); paul.meyfarth@t-online.de (P.M.); merve.saudhof@gmx.de (M.S.); l.van-den-hout@tu-braunschweig.de (L.v.d.H.); jutta.schulze@tu-bs.de (J.S.); r.mendel@tu-bs.de (R.-R.M.); d.kaufholdt@tu-braunschweig.de (D.K.); 2 Center of Molecular Ecophysiology (CMEP), College of Resources and Environment, Southwest University, Tiansheng Road No. 2, Beibei District, Chongqing 400715, China

**Keywords:** *Arabidopsis thaliana*, GUS-staining, hydroponic, localisation, molybdate supply, molybdate transporter, topology, protein–protein-interaction

## Abstract

Molybdate uptake and molybdenum cofactor (Moco) biosynthesis were investigated in detail in the last few decades. The present study critically reviews our present knowledge about eukaryotic molybdate transporters (MOT) and focuses on the model plant *Arabidopsis thaliana*, complementing it with new experiments, filling missing gaps, and clarifying contradictory results in the literature. Two molybdate transporters, MOT1.1 and MOT1.2, are known in *Arabidopsis*, but their importance for sufficient molybdate supply to Moco biosynthesis remains unclear. For a better understanding of their physiological functions in molybdate homeostasis, we studied the impact of *mot1.1* and *mot1.2* knock-out mutants, including a double knock-out on molybdate uptake and Moco-dependent enzyme activity, MOT localisation, and protein–protein interactions. The outcome illustrates different physiological roles for Moco biosynthesis: MOT1.1 is plasma membrane located and its function lies in the efficient absorption of molybdate from soil and its distribution throughout the plant. However, MOT1.1 is not involved in leaf cell imports of molybdate and has no interaction with proteins of the Moco biosynthesis complex. In contrast, the tonoplast-localised transporter MOT1.2 exports molybdate stored in the vacuole and makes it available for re-localisation during senescence. It also supplies the Moco biosynthesis complex with molybdate by direct interaction with molybdenum insertase Cnx1 for controlled and safe sequestering.

## 1. Introduction

Molybdate (MoO_4_^2−^) transport and homeostasis systems in bacteria are quite well understood [1]. Molybdate uptake is facilitated by high affinity systems belonging to the ABC transporter family [2]. The corresponding system in *Escherichia coli* is the modABC operon consisting of three genes *modA*, *modB* and *modC*. ModA is localised in the periplasm and binds molybdate in a highly specific manner. ModB builds an integral membrane channel and ModC is the ATPase subunit in the cytoplasm, energising the process of molybdate transport [3]. In addition, some bacteria use small, cytoplasmatic molybdate binding proteins with an average size of approximately 7 kDA for molybdate storage and homeostasis [4,5].

Soil-living bacteria that perform N_2_ fixation, for example *Azotobacter vinelandii*, have acquired more advanced mechanisms to enable sufficient molybdate supply [6]. As their unique enzyme nitrogenase contains the FeMo cofactor, an efficient acquisition of the essential metals iron and molybdenum is needed [6]. Bacteria release siderophores (high-affinity iron-binding compounds) into their external medium that bind molybdate, in addition to iron. Especially under molybdate limitation, excreted siderophores or “molybdophores” are able to form strong complexes with molybdate, indicating that the availability of molybdate is critical for nitrogenase activity and thus for global nitrogen cycle [7].

In contrast to bacteria, understanding of both the uptake and the homeostasis of molybdate in higher organisms remains incomplete. Especially for plants that rely on nitrate assimilation [8] and thus on the molybdenum dependent enzyme (Mo-enzyme) nitrate reductase (NR), molybdate uptake and distribution throughout the plant body are of great importance. Therefore, NR, as the first enzyme of the nitrate assimilation pathway is of key importance for plant nutrition [9]. To catalyse this essential reaction from nitrate to nitrite, NR requires (besides prosthetic groups FAD and heme) the molybdenum cofactor (Moco) in its active site [10]. All four other Mo-enzymes in plants catalyse essential reactions as well: aldehyde oxidase (AO) is involved in the biosynthesis of the plant hormone abscisic acid and is needed for stomata closure and drought resistance [11]. Xanthine dehydrogenase (XDH) is part of the purine degradation pathway [12]. Mitochondrial amidoxime reducing component (mARC) catalyses the initial N-reduction of amidoximes at the outer mitochondrial membrane in human cells [13], whereas its function in plants is not yet fully understood [14]. The survival of plants in a toxic atmosphere caused by SO_2_ from volcanos or wildfires is only possible with the help of sulfite oxidase (SO) [15,16]. Furthermore, SO plays a role in controlling the cellular concentration of sulfite arising from sulfur metabolism [16]. Therefore, plant growth and development depend on functional Mo-enzymes and their sufficient supply with Moco [17].

To form mature Moco, molybdenum (Mo) is complexed in a highly conserved scaffold called molybdopterin (MPT) where a mononuclear Mo atom is coordinated to a dithiolene moiety [18]. Moco can be found in all kingdoms of life [19]. Like its molecular structure, the Moco biosynthesis pathway is also highly conserved throughout all kingdoms. It involves a complex interaction of six proteins and can be divided into four consecutive steps according to the biosynthetic intermediates of each step [20]. In plants, these enzymes are named according to the Cnx nomenclature (cofactor for nitrate reductase and xanthine dehydrogenase). The first step takes place in the mitochondria, where guanosine triphosphate is converted to cyclic pyranopterin monophosphate (cPMP) by Cnx2 and Cnx3 [21]. Then, cPMP is exported to the cytosol through involvement of ATM3, an ABC-transporter localised in the inner mitochondrial membrane [22]. In the second step, cPMP is converted into MPT by formation of a dithiolene group. This reaction is catalysed by a hetero-tetrameric MPT–synthase complex, consisting of two larger Cnx6 and two smaller Cnx7 subunits [23]. MPT synthase needs replenishment of Cnx7 with sulfur by MPT synthase sulfurase Cnx5 [24]. The last two steps are catalysed by the two different domains of actin-associated molybdenum insertase Cnx1 [25]. First, Cnx1 G-domain (Cnx1G) adenylates MPT [26]. Then, mature Di-oxo Moco is formed by the insertion of Mo and AMP is cleaved by the E-domain of Cnx1 (Cnx1E) [27]. The highly oxygen-sensitive Moco is inserted directly into NR, SO and mARC through direct interaction of Cnx1 with target enzymes as shown in split reporter studies [28], while XDH and AO on the other hand requires sulfurised mono-oxo Moco with a final maturation step catalysed by Moco sulfurase ABA3 [29].

Completion of Moco by Mo-insertase Cnx1 depends on the availability of the oxyanion molybdate (MoO_4_^2−^) which is the only water-soluble and biologically active source of Mo for plants [30]. Molybdate is found in soil, but its bioavailability strongly depends on soil properties such as pH, organic matter content, concentration of adsorbing oxides or the rate of water drainage [31]. In alkaline soils, molybdate becomes more soluble whereas in acidic soils it tends to be unavailable for plants. Therefore, molybdate deficiency more likely occurs in acidic soils (pH < 5.5) due to increased molybdate adsorption to positively charged oxides [17].

It has long been thought that molybdate uptake in plants is facilitated non-specifically by sulphate transporters as sulphate (SO_4_^2−^) and molybdate are highly similar in size, charge, and their tetrahedral structure [32]. In fact, studies with the transporter SHST1 from the legume *Stylosanthes hamata* in yeast cells confirmed that molybdate uptake is facilitated by this sulphate transporter [33]. However, when a group five of sulphate transporters in *Arabidopsis thaliana* (*A. thaliana*) was investigated more closely, it turned out that two members were structurally distinct from the isoforms of the other four groups [34] because they lacked the STAS-domain that is necessary for sulphate transport [35]. Instead of sulphate, one of these group five transporters mediated high affinity molybdate transport (Km ≈ 20 nM) and was therefore renamed to MOT1.1 (AT2G25680, Molybdate Transporter 1.1; formerly named SULTR5; 2 or MOT1). As the *mot1.1* promoter showed expression mainly in roots but also in shoots, it has been suggested that MOT1.1 enhances molybdate uptake from soil into root cells and also enables its translocation into shoots [36]. Studies with *mot1.1* KO grown under molybdate deficiency revealed reduced growth and yellow leaf colour in *A. thaliana* [36,37]. Interestingly, these phenotypes were only observed in the second generation of plants grown under deficiency conditions. Furthermore, an increase in transcript of the Mo-enzyme NR was apparent in these plants [37]. Genome-wide association analyses of 340 natural *A. thaliana* accessions showed that variations in leaf Mo content were strongly associated with polymorphisms in the locus around *mot1.1* gene, indicating its importance for molybdate homeostasis [38]. However, subcellular localisation remains unclear up to this day as the publication by Tomatsu et al. [36] indicated a MOT1.1 presence in the plasma membrane and the endomembrane system whereas Baxter et al. [39] suggested a localisation in mitochondria.

The second member of the molybdate transporter family is MOT1.2 (AT1G80310, formerly named SULTR5;1 or MOT2) with 72% sequence similarity to MOT1.1. It is localised in the tonoplast and fulfils its function as vacuolar molybdate exporter into the cytosol [40]. During senescence, *mot1.2* expression is highly upregulated. Therefore, an important role in inter-organ allocation and relocation of molybdate during seed development was proposed for MOT1.2 [40].

In silico comparative analyses of MOT1 orthologues showed that other plant species and especially legumes have increased numbers of MOT1 family members [41]. For example, soybean (*Glycine max*) has seven members of MOT1 transporters and five were found in *Medicago truncatula* (*M. truncatula*) [42]. The increased number of MOT1s especially in legumes plead for their important role in supplying molybdate for the nitrogenase activity of symbiotic N_2_-fixing rhizobia living in root nodules. The MOT1.1 orthologue of *M. truncatula* *Mt*MOT1.2 was found to be required for molybdate delivery to the endodermis [42]. *Mt*MOT1.3 most likely introduces molybdate into nodule cells where bacterial transporters take up the delivered molybdate to build the essential nitrogenase FeMo cofactor [43]. *Mt*MOT1.2 is located in the endodermal cells of roots and nodules [42], whereas *Mt*MOT1.3 is exclusively located in the plasma membrane of nodule cells [43].

The recently identified *Lj*MOT1 from *Lotus japonicus*, however, seems to have similar functions as MOT1.1 from *A. thaliana*. This plasma membrane localised transporter might be responsible for molybdate uptake from soil and its distribution inside the plant rather than for molybdate delivery to the nodules [44].

Studies on the unicellular algae *Chlamydomonas reinhardtii* revealed the possible presence of two MOT families [45,46]. *Cr*MOT1 [45] mediates the high-affinity transport of molybdate (Km = ~7 nM) comparable with the high affinity transporter *At*MOT1.1. The member of the second family *Cr*MOT2 shows only a low sequence identity of 11–14% to *Cr*MOT1 and its orthologues are present in most eukaryotes, including plants and animals. Yeast cells transformed with *Cr*MOT2 showed a specific molybdate uptake activity (Km = ~550 nM) which is still in the range of high affinity systems [46]. Interestingly, orthologues of *Cr*MOT2 have been reported in animals, like human [46], even though little is known about the MOT2 family in animals. So far, *Hs*MOT2 is the only MOT described in humans with a sequence similarity of 34.5% on amino acid level to *Cr*MOT2 as well as a molybdate transport activity (Km = ~546 nM) after heterologous expression in yeast [46]. *Cr*MOT2 orthologues have also been reported in higher plants such as in *Oryza sativa* [47] and one orthologue (MOT2) has been reported in *A. thaliana* [48], but it was not analysed in detail [49].

It has been shown that molybdate deficiency severely impacts plant growth and development which is mostly the result of reduced Mo-enzyme activity [17]. The knock-out mutants of *mot1.1* show yellow leaves and retarded growth when grown under molybdate deficiency [37]. Both Moco biosynthesis as well as MOT1 family members have been studied intensively. However, the link between MOTs and Moco biosynthesis, including their importance for sufficient molybdate supply remains unclear. In recent years, many biological pathways were found to be highly organized for increased resilience against network disturbances [50]. There are numerous enzymes which only function properly when organised in multi-enzyme complexes [51]. A model was postulated showing that cytosolic Moco biosynthesis enzymes form a biosynthesis complex which is anchored to cytoskeletal elements [25] and that transfer of mature Moco to the apo-Mo-enzymes is carried out via protein–protein interactions [28]. The purpose of the formation of this multi-enzyme complex was proposed to enable both (i) protection of oxygen-sensitive Moco as well as (ii) an efficient substrate channelling [23].

Based on these facts, we hypothesised that the supply of molybdate to Moco biosynthesis could also be realised by a protein–protein interaction between MOTs and Cnx1. This direct substrate channelling could not only accelerate the biosynthesis of Moco, but the postulated direct transfer could also be an important part of metal homeostasis to protect cells from the toxic effects of free heavy metal ions [52]. This aimed to determine the different functions of the MOT1 family members regarding their importance for molybdate homeostasis and the Moco biosynthesis machinery. Therefore, (i) protein localisation and interactions were studied, (ii) the impact of molybdate transporter knockouts, including a *mot1.1 mot1.2* double knock-out on molybdate uptake and Mo-enzyme activity was demonstrated, and (iii) previously published results of MOT1 family members were summarised to fill missing gaps and to clarify contrary results of previous publications.

## 2. Results and Discussion

### 2.1. Localisation and Topology Studies

The precise function for MOT1.1 from *A. thaliana* is still a matter of discussion because of contradicting publications regarding its subcellular localisation. Two different localisation approaches were described in which MOT1.1 was fused to the green fluorescent protein (GFP) in either N- or C-terminal orientation. Baxter et al. [39] reported a mitochondrial localisation for the MOT1.1-GFP construct in *A. thaliana* protoplasts and roots (co-localised with a fluorescent mitochondrial tracker molecule), whereas Tomatsu et al. [36] showed primarily a localisation in the endomembrane system after expression of GFP-MOT1.1 in tobacco BY-2 cells. It was argued that these discrepancies occurred because a predicted N-terminal mitochondrial targeting signal was supposedly blocked in the GFP-MOT1.1 construct which, therefore, must have led to mis-localisation to the endomembrane system. However, a mitochondrial localisation was also discussed critically by Baxter et al. [39] due to the missing of a comprehensible physiological role of MOT1.1 in the mitochondrial membrane.

Therefore, we attempted to reinvestigate this contradictory localisation of MOT1.1 and began with an in silico analysis. The ARAMEMNON database, originally described by Schwacke et al. [53] and since regularly updated, combines results of different analyses algorithms and compares predictions of 20 different localisations. In summary, only 3 out of 20 tools suggest a localisation of MOT1.1 in a mitochondrial membrane (score: 3.7). Furthermore, a localisation in the nucleus is almost as highly predicted as a mitochondrial localisation (score: 3.5). Only 1 out of 20 tools predicted a localisation in the secretory pathway (score 1.4) and some tools even suggested a chloroplast localisation (score 1.1). Therefore, the MOT1.1 localisation prediction is ambiguous.

We started with an experimental approach in *N. benthamiana* using a Venus-MOT1.1 construct with an N terminally fused yellow-fluorescent protein, analogous to the experiment of Tomatsu et al. [36]. The result confirmed a strong fluorescence at the outermost rim of the cell that was clearly distinct from the eqFP611 cytosolic marker (Figure S1A). However, a noticeable Venus fluorescence was detectable in the cytoplasm and the nucleus which might originate from incomplete fusion proteins. Nevertheless, the N-terminal fusion construct indicated a plasma membrane localisation in *N. benthamiana* leaf cells as described by Tomatsu et al. [36]. However, this N-terminal fusion orientation appears to be unusable as expected by Baxter et al. [39] because it seems to block targeting signals.

**Figure 1:**
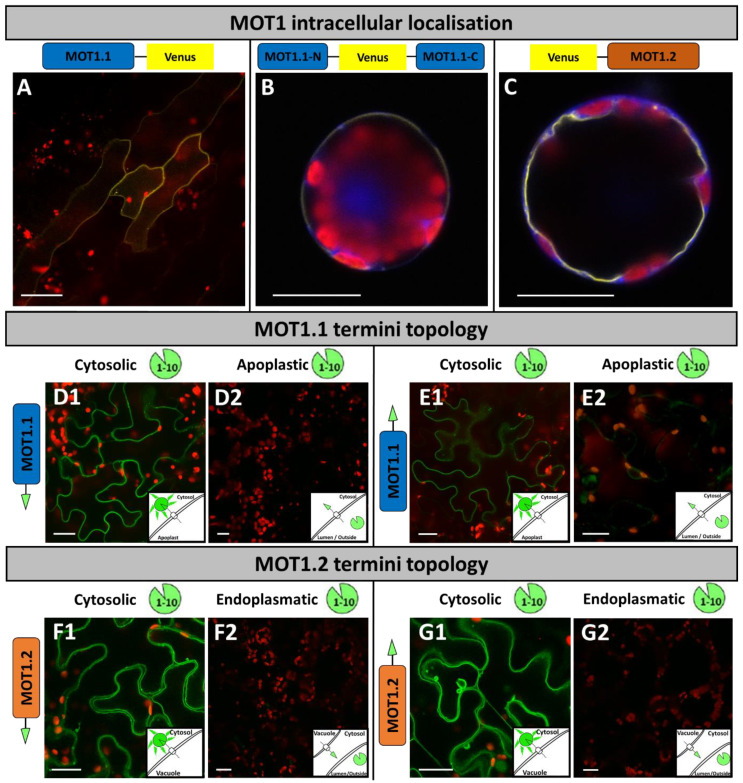
**Intracellularlocalisation of MOT1 family members and termini topology.** Localisation studies of MOT1 family members (**A**–**C**) were performed by transient transformation of (**A**) *A. thaliana* via FAST method, and (**B**,**C**) *N. benthamiana* mesophyll protoplasts via chemical transformation to express fusion proteins of Venus and MOT1.1 in different orientations. *N. benthamiana* protoplasts were co-transformed with cytosolic marker eqFP611. Images were merged from (**A**) Venus, chlorophyll auto-fluorescence channels, and also (**B**,**C**) with the eqFP611 fluorescence channel. Split-GFP topology studies (**D**–**G**) were carried out with transient transformation by *Agrobacterium* infiltration of *N. benthamiana* leaves to express GFP11-MOT fusion proteins. Two days later, leaves were co-transformed with GFP1-10 (**D1**,**E1**,**F1**,**G1**); SP-GFP1-10 (**D2**,**E2**); and SP-GFP1-10-HDEL (**F2**,**G2**). Images were taken 2–3 days after transformation and merged from GFP and chlorophyll auto-fluorescence channels. Each image was taken with a C-Apochromat 40×/1.2 water immersion objective and scale bars depict a length of 20 μm.

Consequently, the C-terminal fusion construct was analysed. The transient heterologous expression of MOT1.1-Venus in *N. benthamiana* supported a localisation in a network-like endomembrane system (Figure S1B). In contrast, specific fluorescence in the homologous system of *A. thaliana* seedlings was detected as a thin uniform layer along the cell surface of epidermis cells, and chloroplasts were not surrounded by fluorescence in these cells (Figure 1A). In conclusion, MOT1.1 is localised in the plasma membrane. Since this result differed from the observations of Baxter et al. [39], who used the same C-terminal fusion orientation, we designed an additional fusion construct where the Venus reporter is located in a huge loop between the third and fourth intermembrane domains of MOT1.1 (MOT1.1N-Venus-MOT1.1C). This construct should enable a MOT1.1 localisation without blocking putative targeting signals at the N- or C-terminus. In *N. benthamiana* mesophyll protoplasts, the fluorescence of MOT1.1N-Venus-MOT1.1C encircled the protoplast at its periphery, and a clear differentiation to the co-expressed fluorescent cytosol marker was observed (Figure 1B and Figure S1E). Interestingly, no aggregations in the endomembrane system around the nucleus were observed here, indicating that the protein-internal Venus reporter did not lead to an artificial remaining in the endomembrane system. Co-localization studies of MOT1.1N-Venus-MOT1.1C and the plasma membrane marker SCFP-AtPIP2a in the epidermis cells of *A. thaliana* showed a perfect overlay of both signals at the outer most periphery of the cell when merged (Figure S1C). Taken together, our results support the plasma membrane localisation of MOT1.1.

The tonoplast localisation of MOT1.2 was identified without doubt by Gasber et al. [40], who used a MOT1.2-GFP construct expressed in onion cells and *A. thaliana* protoplasts. Because of the discrepancies of the MOT1.1 localisation in previous publications due to different reporter/transporter fusion orientations, we aimed at verifying the tonoplast localisation by transforming the N-terminal fusion construct Venus-MOT1.2. As observed previously by Gasber et al. [40], the fusion construct is localised in the tonoplast, identifiable by the thin layer of fluorescence along the inner side of the cytosol and organelles such as chloroplasts (Figure 1C and Figure S1F). Performed co-localization studies with MOT1.2-Venus and the plasma membrane marker SCFP-AtPIP2a showed no overlay of the signals in the epidermis cells of *N. benthamiana* (Figure S1D). MOT1.2-Venus was localized in proximity to SCFP-AtPIP2a, leaving space for a thin cytoplasm tube indicating tonoplast localization. Furthermore, the nucleus is surrounded first by a signal of SCFP-AtPIP2a fluorescence and secondly by MOT1.2-Venus fluorescence. Therefore, MOT1.2 was clearly verified as a membrane protein of the tonoplast.

As a next step, topology studies were performed to determine the orientation of N- and C-termini which is of great importance, especially when it comes to tag-based protein–protein interaction studies of membrane integrated proteins. First, in silico analyses with the ARAMEMNON database were performed, which compares 18 different topology prediction tools and combines all extracted information to produce a consensus. The number of predicted transmembrane domains (TM) varied for MOT1.1 from 7 to 12. Extended consensus prediction resulted in 10 TMs, where consensus predictions of several homologous proteins were also taken into consideration. The predicted number of TMs for MOT1.2 varied from 6 to 11 with a consensus of 10 TMs. Accordingly, the orientation of the termini differed between the algorithms.

Therefore, we investigated the topology experimentally by a split-GFP system [54]. The 11th β-sheet of GFP (GFP11) was fused to the MOT1 family members N- or C-terminally. The corresponding GFP1-10 construct was co-transformed in *N. benthamiana*. It localised either in the cytosol (GFP1-10), in the apoplast (SP-GFP1-10), or was retained in the endoplasmic reticulum (SP-GFP1-10-HDEL) by using respective signal peptides. Only when GFP1-10 and the transporter terminus fused with GFP11 were present in the same compartment fluorescence of the reconstituted GFP was detectable.

When GFP11-MOT1.1 or MOT1.1-GFP11 was co-expressed with cytosolic GFP1-10, GFP fluorescence was observed as a thin uniform layer along the cell surface, and chloroplasts were not surrounded by fluorescence, thus additionally supporting plasma membrane localisation (Figure 1D1,E1). Co-expression of these constructs with apoplastic GFP1-10 (SP-GFP1-10) resulted in a few fluorescing cells with weak accumulations of fluorescence close to the nucleus which is interpreted as overexpression artefacts (Figure 1D2,E2). From these results, it can be concluded that both termini have a cytosolic orientation.

MOT1.2 topology analyses showed that both the N- and C-terminus of MOT1.2 are localised in the cytoplasm, since self-assembly to a complete GFP only occurred when cytosolic GFP1-10 was co-expressed (Figure 1F1,G1). Vacuolar bulbs and transvacuolar strands consisting of a vacuolar membrane further supported tonoplast localisation. When endoplasmatically localised GFP1-10 (SP-GFP1-10-HDEL) was co-expressed (Figure 1F2,G2), only very low levels of fluorescence were detected. Taken together, these results indicate that both termini of MOT1.2 reach into the cytoplasm. These are perfect preconditions for interaction studies. Consequently, both MOT1 family members seem to have an even number of transmembrane domains.

### 2.2. Organ Specific Expression Pattern

When resolving MOT1.1 localisation in the plasma membrane, we attempted to elucidate the physiological role of both MOT1.1 and MOT1.2 for molybdate homeostasis. Their temporal as well as their spatial expression are important indications to distinguish between their function in different developmental stages and organs. For *mot1.1*, previous publications analysed in detail the organ specific expression pattern. However, data for *mot1.2* are still missing. Therefore, we completed the dataset, both for *mot1.1* and *mot1.2*, which was necessary for a clear classification of their physiological functions.

Histochemical experiments were performed by Tomatsu et al. [36] with transgenic *A. thaliana* plants expressing the reporter enzyme GUS under control of the *mot1.1* promoter, defined as 2903 bp upstream of the start codon. In seven-day old seedlings, GUS activity was observed in the mature portion of the root. Plants in the reproductive stage showed GUS activity in the mesophyll and petioles of the leaves. Furthermore, activity was measured in the stamen, calyx and siliques. Baxter et al. [39] performed analogous gene expression experiments. However, they defined the promoter within a region of 1800 bp upstream of the ATG. In adult plants, they observed strong GUS activity in primary and lateral roots, pronounced directly behind the root tip. Cross-sections of roots revealed a distinct restriction to protodermal cells. In the radicular elongation zone, GUS activity was restricted to the epidermis and cortex, but also to the vascular tissue. Fully expanded leaves showed a distinct and strong GUS activity exclusively in the vascular tissue.

**Figure 2:**
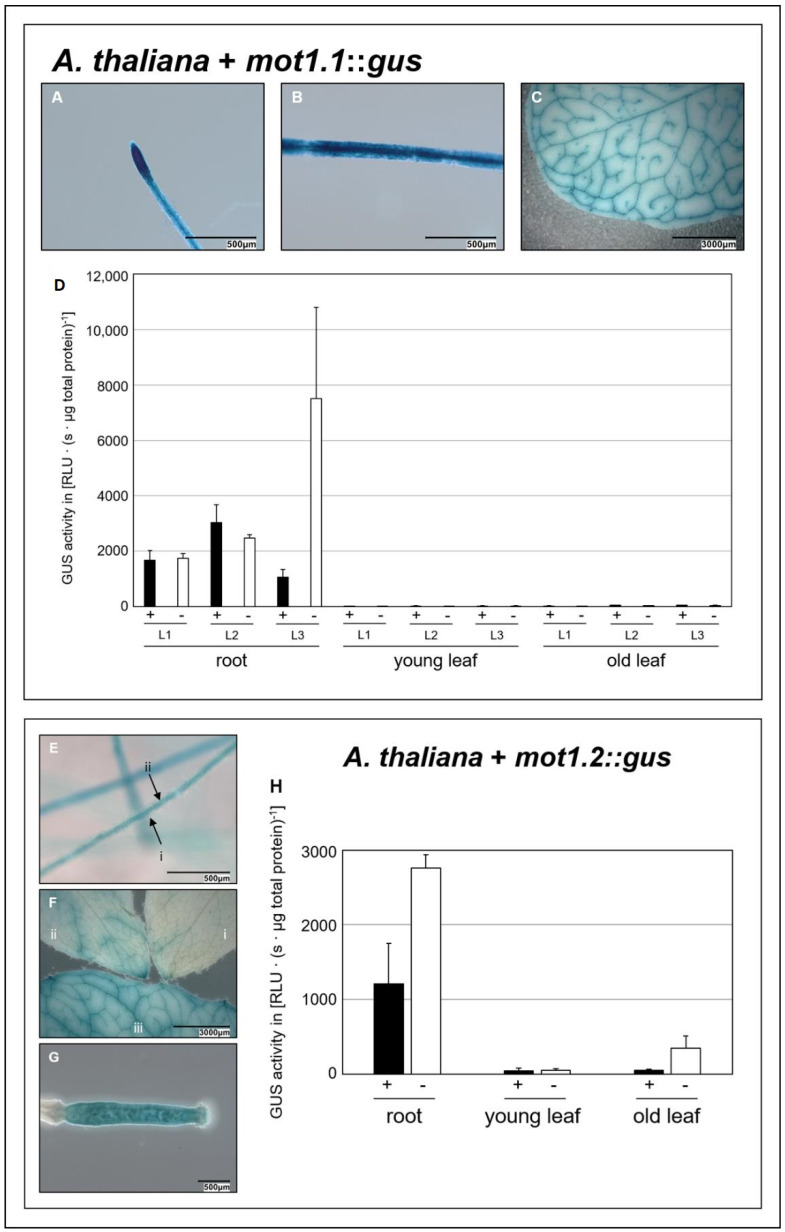
**Organ specific expression patterns of *mot1* family members.** Histochemical GUS assay of transgenic *A. thaliana* carrying *mot1.1::gus* (**A**–**C**). Root tip (**A**), mature root (**B**), and old leaf (**C**) are shown. Histochemical (**E**–**G**) GUS assay of transgenic *A. thaliana* carrying *mot1.2::gus* showing mature root ((**E**); i = unstained root periphery, ii = stained central cylinder), rosette leaves of increasing age ((**F**); (i) young, (ii) middle-aged, (iii) old) and developing fruit (**G**). Scale bars = 500 µm (**A**,**B**,**E**,**G**) and 3000 µm (**C**,**F**). Fluorimetric GUS assay (**D**,**H**) showing GUS activity of three independent (L1–L3) *mot1.1::gus* plant lines and one *mot1.2::gus* line grown with molybdate availability (+) and deprivation (−) in roots, young and old leaves.

In this study, a histochemical staining of *mot1.1*::*gus* lines was included to allow for a direct comparison with analogous stainings of *mot1.2::gus* plants. Analogously to the results of Baxter et al. [39], a strong GUS expression was observed in roots of *mot1.1::gus* plants, with a more prominent concentration in the root tips (Figure 2A) and central cylinder (Figure 2B). Furthermore, adult leaves also showed a very intense and distinct GUS expression in vascular tissues (Figure 2C). In addition, a corresponding fluorimetric GUS assay of different plant organs was performed, enabling quantitative evaluation. High levels of GUS expression in roots (Figure 2D) were detected that appeared to be independent of molybdate availability. Even though GUS expression in mature leaves was visible in the histochemical GUS staining, no signals could be measured in the fluorimetric GUS assay. This points to roots as the main organ of *mot1.1* expression, thus leading to the conclusion that MOT1.1 seems to play only a minor role in importing molybdate into leaf cells.

In the present study, organ specific *mot1.2* expression was analysed for the first time using *mot1.*2::*gus* lines. A strong GUS signal was observed in the central cylinder of roots in the histochemical staining (Figure 2E) as well as in the quantitative fluorimetric assay (Figure 2H). This suggests that MOT1.2 plays an important role in the molybdate distribution in plants, even though the signal can be considered to be lower than the expression level of *mot1.1,* which might speak for a more supportive role of MOT1.2. Histochemical staining of leaves also revealed a correlation between *mot1.2* expression and increasing age (Figure 2F). This result is in full congruence with observations made by Gasber et al. [40]. They detected an increased mRNA level during senescence when using a northern blot analysis. Furthermore, they observed a significantly reduced amount of molybdate in the seeds of *mot1.2* KO plants and concluded that MOT1.2 is an exporter of molybdate from senescent tissue, making it available for seed loading during maturation.

The histochemical staining of the present study showed a strong GUS-activity in fertilised ovaries where seed development takes place (Figure 2G). All taken together, strong and exclusive expression of *mot1.2* in fertilised ovaries suggests not only the export function of molybdate from senescent leaves, but also its active involvement in the loading of seeds. Interestingly, the expression of *mot1.2* was increased under molybdate depletion in roots and old leaves (Figure 2H). The fact that a certain degree of molybdate shortage in plants induced *mot1.2* expression in leaf tissue to increase vacuolar molybdate exports into the cytosol underlines the importance of MOT1.2 for an effective Moco biosynthesis.

### 2.3. Macroscopic and Molecular Phenotype Analyses Reveal Different Roles of MOT1 Family Members for Moco Biosynthesis

The phenotype of Mo deficiency in plants of the *Brassicaceae* family ranges from mottling and flaccid leaves to dwarfism until dying and is consistent throughout different species. Tomatsu et al. [36] studied the *mot1.1*KO line (SALK118311) in a rockwool system. Plants that grew under molybdate limitations showed severely reduced growth in both shoots and roots. Further phenotypical signs of molybdate deficiency were not reported. Ide et al. [37] used the same plant line for comparable phenotype analysis and reported retarded growth and yellowish leaf colour. Gasber and colleagues [40] analysed the impact of molybdate deprivation on a *mot1.2* KO line (SALK_015044), but could not observe an altered phenotype when grown on soil or under molybdate limitation conditions in a liquid growth setup.

The present study aims to grow both lines (Table 1) under the same molybdate limitation conditions in a hydroponic growth system (modified according to [55]) for comparative analysis. For the first time, we also included a *mot1.1-mot1.2-*double knock-out (*mot1.1 mot1.2* dKO, Table 1) that was kindly provided by the research group of Professor Ekkehard Neuhaus (TU Kaiserslautern, Germany). With this, we aim to gain a comprehensive insight into the function of the MOT1 family and their interplay to maintain the global molybdate homeostasis in planta.

**Figure 3:**
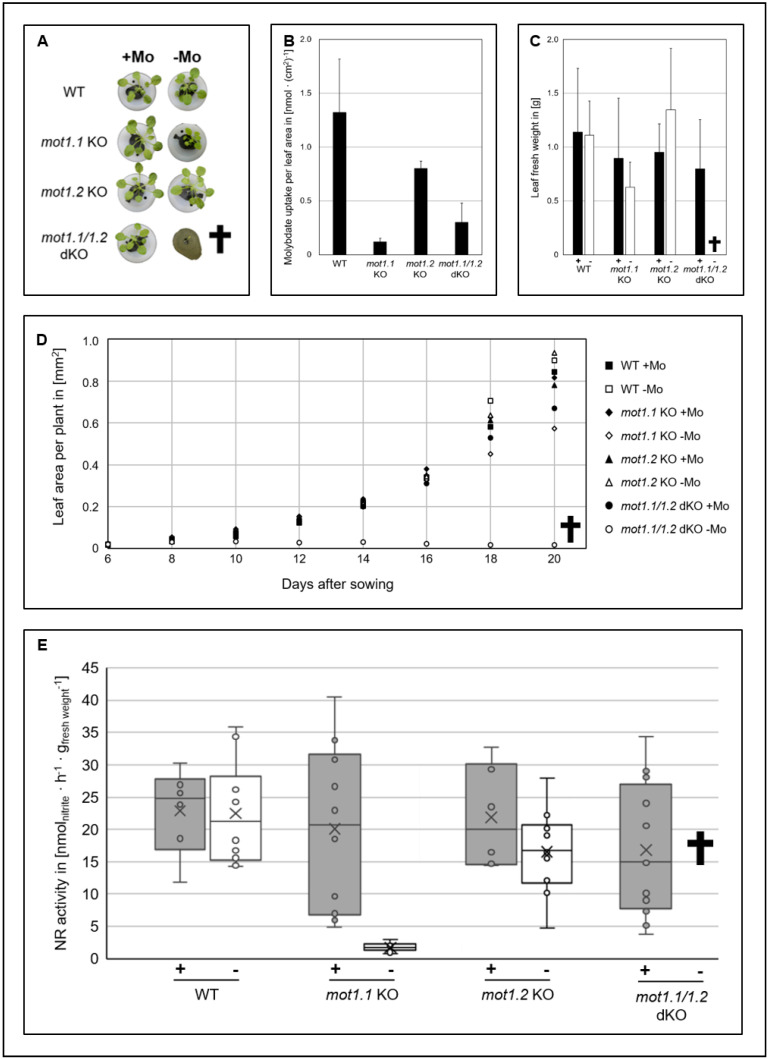
**Impact of molybdate deprivation on macroscopic and molecular phenotype on *A. thaliana mot1* KO lines.***Arabidopsis* KO lines were grown hydroponically under molybdate availability (+Mo) and molybdate deprivation (−Mo) conditions. (**A**) Macroscopic phenotype 34 days after sowing. (**B**) Molybdate uptake per plant normalised to leaf area. (**C**) Rosette fresh weight 60 days after sowing. (**D**) Leaf area per plant during growth period of 20 days. (**E**) NR activity of plants grown under molybdate availability (+) and deprivation (−). Boxplot: X = mean, middle line = median. **†** = *mot1.1 mot1.2* dKO died under –Mo conditions, not allowing the performance of further analysis.

Interestingly, wild type plants did not show altered growth behaviour (Figure 3A,C,D) when facing molybdate deprivation conditions. Due to this observation, the molybdate concentration of the basic nutrient solution (BNS) without molybdate supplementation was determined in triplicates according to the modified protocol of Cardenas and Mortensen [56] and showed a concentration below 10 nM. This suggests that a minimal molybdate concentration caused by contaminations deriving from other components of BNS without an additional molybdate supplementation is already sufficient to allow growth such as under full molybdate availability (c = 100 nM) conditions. In conclusion, this illustrates two things: (i) the demand of molybdate in *A. thaliana* is very low, and (ii) *A. thaliana* possesses highly effective and highly affine molybdate transporter systems to cope with a minimal molybdate concentration.

When grown under molybdate deprivation, *mot1.1* KO showed a pale green leaf colouration with partially necrotic areas (Figure 3A) and a reduced growth rate (Figure 3D), culminating in a reduced fresh weight after 60 days when plants were harvested (Figure 3C). Taken together, the present study shows a phenotype comparable to that described by Tomatsu et al. [36] with even further signs of molybdate deprivation as described by Ide et al. [37]. Interestingly, this phenotype was not observed under molybdate availability. The described phenotypic characteristics are mainly caused by a general deprivation of reduced nitrogen compounds due to the reduced activity of the Mo-enzyme NR [17]. An additional nitrate fertilisation can attenuate this phenotype to a pale green leaf colouring accompanied by small necrotic regions and an overall reduced growth rate [17].

It is also of interest to point out that molybdate deficiency phenotypes described in the present study already occurred in the first generation of *mot1.1* KO plants grown in hydroponics. Both Tomatsu et al. [36] and Ide et al. [37] reported a phenotype only in the second generation of plants grown under molybdate deficiency. This further underlines the severe molybdate deprivation achieved with hydroponics in the present study.

Taken all together, the observations clearly show that MOT1.1 is a high-affinity importer of special importance when it comes to molybdate limitation, but it can also be compensated by other mechanisms when molybdate is abundant. The *mot1.2* KO did not show differences in growth behaviour under both conditions in comparison to wild type (Figure 3A,C,D). This is consistent with the observations of Gasber and colleagues [40].

The most striking effect of molybdate deprivation was observed for the *mot1.1 mot1.2* dKO. The seeds were poorly germinated and the plants suffered from severe dwarfism (Figure 3A,D). Within 22 days after sowing, the whole population of dKO plants grown under molybdate deprivation (*n* = 48) died, while all other plant lines with single knock-outs described above reached survival rates near 100% under molybdate deprivation. These findings underline the crucial role of a functional interplay between the members of the MOT1 family for plant survival under limited molybdate availability. Interestingly, *mot1.1 mot1.2* dKO did not show an altered phenotype compared to the wild type under sufficient molybdate. This observation suggests that the survival of *mot1.1 mot1.2* dKO under molybdate availability is potentially mediated by members of the MOT2 family functioning as backup for the MOT1 family.

Molybdate uptake from substrate is facilitated by the plant root. Even though the mechanism of molybdate uptake into roots is not fully understood, MOT1.1 clearly plays an important role in molybdate uptake especially under molybdate limitation. Investigating whether the other members of the MOT1 family, namely MOT1.2, are also somehow involved in molybdate uptake or share a functional cooperation between both members of the MOT1 family is crucial for plant vitality. As such, molybdate uptake assays of dedicated KO lines were performed.

In the present study, the *mot1.1* KO plants showed a molybdate uptake rate of only 10% of the wild type (Figure 3B). This observation matches the described reduction in molybdate in roots and shoots of the same KO line grown either on soil [36], solid media [37] or hydroponically [39], and it underlines the assumed role of MOT1.1 as a main molybdate importer in *A. thaliana*. Molybdate uptake rate of *mot1.2* KO stands in particular discrepancy to the observations made by Gasber and colleagues [40]. They described no changes in overall molybdate concentration in extracts of whole rosettes, whereas highly increased molybdate levels in withered and dried rosette leaves compared to the wild type were measured. In addition, a reduced molybdate concentration in mature seeds was observed. Our data indicate a reduced molybdate uptake activity by 40 % compared to the wild type (Figure 3B). Furthermore, the *mot1.1 mot1.2* dKO showed molybdate uptake rates comparable to the *mot1.1* single KO, implying that MOT1.1 plays the main role of molybdate uptake in *A. thaliana* and an additional KO of *mot1.2* does not further decrease molybdate uptake.

However, measurement of molybdate uptake does not suffice if we want to understand the physiological role of the MOT1 family. Besides sufficient uptake and distribution throughout the plant an effective supply of Moco biosynthesis with molybdate by at least one member of MOT1 family is necessary. Appropriate insights can be gathered by determining the activity of Moco-user enzymes such as NR.

The median of NR activity is reduced by 18% in the WT when comparing molybdate limitation to sufficient molybdate availability (Figure 3E). This indicates that the wild type suffers from molybdate deprivation stress; however, not to the extent that would manifest in an altered phenotype or a reduced growth rate. Interestingly, *mot1.1* KO shows a slightly reduced NR activity under molybdate availability compared to the wild type. This underlines that the plants already face a certain degree of molybdate deprivation stress under full molybdate availability. However, the level of stress appears to be insufficient to result in altered growth behaviour. Under molybdate limitation, the NR activity of *mot1.1* KO is severely reduced by roughly 90%. This indicates that reduced molybdate uptake rate observed in *mot1.1* KO has a severe impact on Moco biosynthesis, resulting in a strongly reduced NR activity. In *C. reinhardtii* it was observed that decreased molybdate uptake rates caused by interferences in *Crmot1* expression affected NR activity underline the relationship between nitrate reduction and molybdate availability [45]. Interestingly, Ide et al. [37] reported highly increased transcript levels of both *nia* genes (*nia1* and *nia2*) in *mot1.1* KO of *A. thaliana* grown under molybdate deficiency. They concluded an induction of *nia* expression due to a molybdate and consequently Moco, shortage in these plants to compensate for reduced nitrate assimilation. In the present study, drastically decreased NR activity levels in *mot1.1* KO plants grown under comparable conditions were observed, thus supporting this conclusion. Furthermore, it can be assumed that the observed molybdate deprivation phenotype is mainly caused by a lack of reduced nitrogen compounds due to the loss of NR activity [17]. Interestingly, *Nicotiana tabacum* showed lower plant growth rates only when NR activity was reduced to 10% [57], an activity level comparable to *mot1.1* KO under molybdate deprivation conditions as observed in the present study. This underlines the fact that the reduced NR activity causes the reduced growth rate of the *mot1.1* KO.

Gasber and colleagues [40] observed a reduced NR activity in *mot1.2* KO under both molybdate availability and molybdate limitation conditions to a level of 75% and 25%, respectively, when compared to the wild type under the same conditions. Interestingly, the overall Mo amount in the plants increased [40]. In the present study, the *mot1.2* KO showed a comparable reduction under molybdate availability to a level of 65% of wild type NR activity. Under molybdate deprivation conditions a decrease in NR activity by roughly 20% was observed in these plants.

MOT1.2 is localised in the tonoplast and a loss of the transporter was found to lead to a slight increase in vacuolar molybdate [40]. In conclusion, it is postulated that MOT1.2 is involved in the vacuolar export of molybdate into the cytosol. Therefore, the observed reduction in NR activity is in complete congruence to these observations. Loss of MOT1.2 leads to a lock up of molybdate in the vacuole which, in consequence can no longer be made available for Moco biosynthesis. The lack of Moco leads to a reduced NR activity, whereas the overall molybdate content in the cell is increased. 

The NR activity of *mot1.1 mot1.2* dKO under molybdate availability is comparable to the *mot1.1* single KO, suggesting that a potential MOT backup system transports molybdate in an amount that maintains a stable level of NR activity and, more importantly, is independent from members of the MOT1 family. Then, severe dwarfism followed by the death of *mot1.1 mot1.2* dKO under molybdate limitation did not allow the analysis of NR activity. Interestingly, the *mot1.1 mot1.2* dKO grown under a moderate molybdate availability of 50 nM did not show an altered macroscopic phenotype compared to full molybdate availability (Figure S3).

The present study underlines the assumed role of MOT1.1 as the main radicular importer of molybdate into the plant. The role of MOT1.2 clearly appears to be the main exporter of molybdate from the vacuole where molybdate is stored.

### 2.4. Interplay of MOT1s with Proteins of Moco Biosynthesis

Many biosynthesis pathways were discovered to be highly organised which guarantees a safe and efficient substrate channelling [50]. In this context, cytosolic Moco biosynthesis proteins Cnx5, Cnx6, Cnx7 and Cnx1 were found to undergo tight protein interactions in a multi-protein complex in *A. thaliana*. In addition, Cnx1 anchors this complex on actin filaments [25]. In that way, oxygen sensitive Moco intermediates are protected during biosynthesis through micro-compartmentalisation which enables an efficient and degradation-free mechanism [20]. In the last step of Moco biosynthesis, molybdate is inserted into the pterin-based backbone (MPT) by the two-domain protein Cnx1 [58].

In order to do so, not only does MPT need to be transferred to Cnx1, but molybdate must also be directly available. However, as Mo is a heavy metal, it might negatively affect some cellular processes as it is known to do with other heavy metals such as cadmium or copper. These metals underlie the complex regulatory mechanisms to prevent toxic effects [59]. The fact that molybdate is nonetheless needed for Moco biosynthesis is, however, challenging for plant cells. A balance must be kept between keeping the concentration of free molybdate as low as possible and as abundant as needed. Accordingly, a protein–protein interaction between Cnx1 and MOT1 transporters to maintain molybdate homeostasis was hypothesized in previous studies [60,61], but never shown experimentally. MOT1.1 seems to act as a high affinity molybdate importer and can supply Cnx1 from the outside of the cell. However, MOT1.2 can also transport molybdate from the vacuolar storage. Decreased NR activity in *mot1.2* KO plants [40] and the increased expression of *mot1.2* under molybdate deprivation are valid hints speaking for the role of MOT1.2 as the supplier of Cnx1 with molybdate.

To elucidate the interaction network between MOT1s and Cnx1, protein interactions were studied via bimolecular fluorescence complementation (BiFC). Based on topology studies, it was known that both termini extend into the cytosol. This enabled us to use both N- and C-terminal reporter fusions for interaction studies. Negative control proteins need similar characteristics compared to the protein of interest. Therefore, plasma membrane localised aquaporin PIP2a [62] and tonoplastic inositol transporter INT1 [63] were used as negative controls for the corresponding MOT1-family members. Additional abundance controls guaranteed an equal concentration of the protein of interest and the negative control in cells to avoid the effects of random interaction [28].

**Figure 4:**
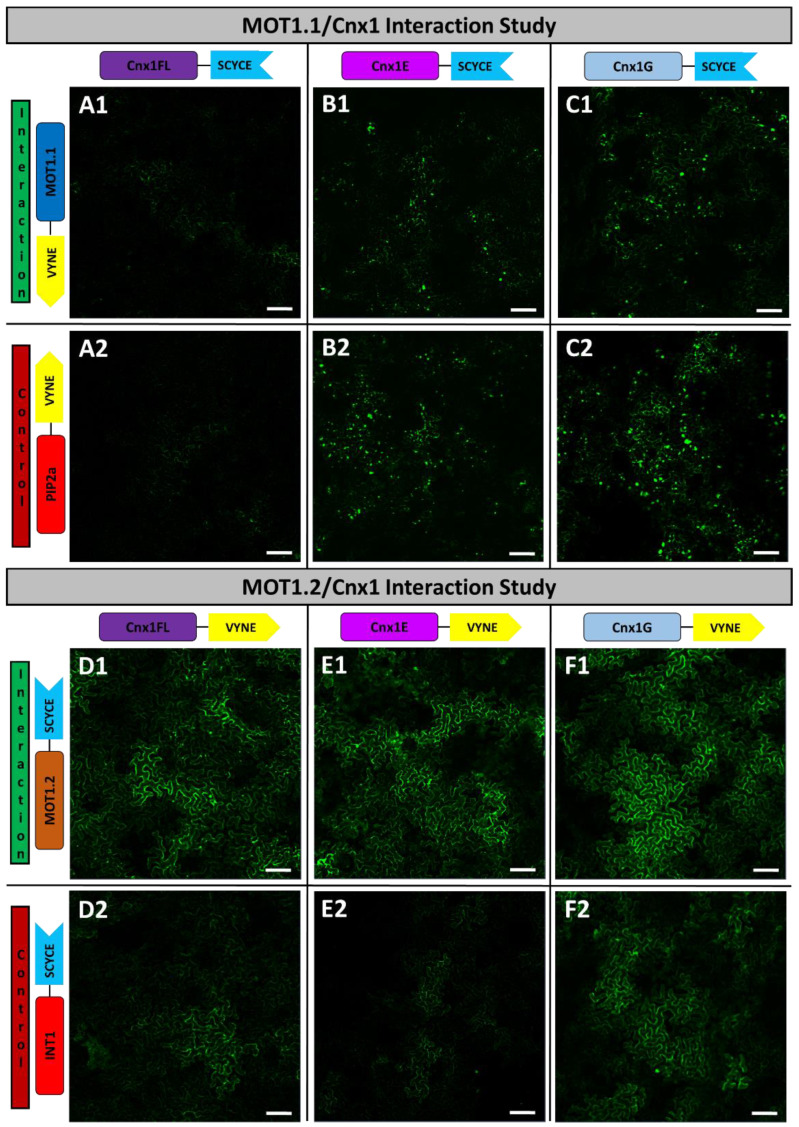
**Interaction studies of MOT1 family members and Cnx1 via BiFC.** Interaction approaches showing transiently transformed leaves of *N. benthamiana* co-expressing VYNE-MOT1.1 and Cnx1-SCYCE (**A1**): Cnx1 full-length (FL)-SCYCE, (**B1**): Cnx1 E-domain (Cnx1E)-SCYCE, (**C1**): Cnx1 G-domain (Cnx1G)-SCYCE). In the negative controls (**A2**,**B2**,**C2**), PIP2a-VYNE replaces VYNE-MOT1.1. Analogous interaction approaches with MOT1.2-SCYCE and Cnx1FL-VYNE (**D1**), Cnx1E-VYNE (**E1**) and Cnx1G-VYNE (**F1**) are shown. In the negative controls (**D2**,**E2**,**F2**), INT-SCYCE replaces MOT1.2-SCYCE. Images were taken with a Plan-Neofluar 10×/0.3 and scale bars depict a length of 100 μm. Corresponding abundance controls where NSP3 replaces Cnx1, are shown in Figure S4.

In the first interaction approach, reporter fragment fusion construct VYNE-MOT1.1 was co-expressed with full-length Cnx1 (Cnx1FL) that was fused C-terminally to the corresponding BiFC reporter fragment SCYCE (Cnx1FL-SCYCE). No interaction was observed as fluorescence intensities were on equal levels between the interaction approach (Figure 4A1) and negative control (Figure 4A2). Visible aggregates are based on the ability of Cnx1 to form multimeric complexes due to overexpression as observed in previous studies [20]. In a subsequent approach, other fusion orientations of both Cnx1FL and MOT1.1 were tested to eliminate the possibility of steric hindrance and therefore the misinterpretation of previous results. However, no interactions were detected (Figure S2). Additionally, two separately expressed domains of Cnx1 were tested with MOT1.1. Taking all the results together, an interaction of MOT1.1 with the E-domain as well as with the G-domain of Cnx1 could not be shown with BiFC experiments (Figure 4B,C).

The following experiments were conducted analogously with MOT1.2. As a first interaction approach, MOT1.2-SCYCE was co-expressed with Cnx1FL-VYNE. In direct comparison, fluorescence intensity in the interaction approach appeared much brighter (Figure 4D1) than the negative control with INT1-SCYCE (Figure 4D2). Again, both the E- as well as the G-domain were tested to identify the interacting domain. Both domains showed higher fluorescence intensities when co-expressed with MOT1.2, compared to the negative control. While the observed fluorescence of Cnx1G was only slightly brighter (Figure 4F), the fluorescence with Cnx1E differed significantly in comparison to the negative control. The result was verified with another fusion orientation (Figure S3). Thus, the increased fluorescence intensities observed in our interaction approaches indicate the direct interactions of MOT1.2 with the E-domain of Cnx1 which mediates the molybdate insertion into MPT-AMP. Therefore, MOT1.2 seems to supply Cnx1 with molybdate from the vacuolar storage for Moco biosynthesis.

## 3. Conclusions: Both MOT1 Family Members Have Different Physiological Roles for Molybdate Supply of Cnx1

The present study completes the characterisation of *A. thaliana*’s MOT1 family members and sheds light on their different physiological functions by summarising knowledge from several previous studies and combining this with new data. In addition, a novel link to the Moco biosynthesis complex was introduced via BiFC interaction studies.

MOT1.1 was verified to be the main root importer of molybdate into the plant. Loss of this transporter leads to severely reduced growth and a molybdate deficiency phenotype under molybdate limitation, appearing as a pale green-yellowish leaf colour and partially necrotic tissue. The *mot1.1* KO showed by far the lowest level of molybdate uptake which presumably led to an impaired Moco biosynthesis rate, resulting in a decrease in NR activity. In addition, intracellular localisation was clarified in the plasma membrane, which is a necessary precondition for it to function as a high affinity importer. However, expression that is exclusive to roots and vascular tissue indicated no direct involvement in importing molybdate into the cells of leaf tissue and consequently, it showed no direct role in supplying the Moco biosynthesis complex with molybdate. This indication is supported by the lack of interaction with Mo-insertase Cnx1.

MOT1.2 appears to be the main exporter of molybdate from its storage in the vacuole. This can be supported by a precise localisation in the tonoplast as well as a slightly increased vacuolar molybdate concentration in KO plants [40]. Loss of MOT1.2 leads to a lock up of molybdate in vacuoles causing reduced NR activity under molybdate deprivation. Furthermore, through the results of our BiFC interaction studies we identified MOT1.2 to be the molybdate supplier for Moco biosynthesis. The involvement of MOT1.2 in Moco biosynthesis was also speculated by Gasber et al. [40] as they observed an enrichment of the intermediate MPT in the *mot1.2* KO. We conclude that molybdate is probably transferred directly to Mo-insertase Cnx1 for an efficient Moco-biosynthesis process. In addition, this interaction prevents the free diffusion of heavy metals into the cytoplasm of plant cells.

Next to its role in metabolically active tissue, MOT1.2 plays an important role in senescence. The transcription level [40] and the activation of the *mot1.2* promoter are linked to leaf age. With the increasing age of the leaf, MOT1.2 is activated and exports the remaining molybdate from the vacuoles for interorgan molybdate translocation [40]. Besides this, MOT1.2 also plays an important role in fertilised ovaries due to its local strong and distinct expression. Two potential necessities for an increased vacuolar molybdate export in this organ may be envisaged: (i) abscisic acid (ABA) is synthesised by the Mo-enzyme AO [11] and a sufficient accumulation of the phytohormone in maturing seed is responsible for dormancy during its development [64]. Besides ABA derived from the embryo itself, ABA derived from maternal tissue such as the testa participates to the status of dormancy as described in *A. thaliana* [65]. In conclusion, the generation of elevated ABA levels in both maternal and embryonic tissue requires an increased activity of AO in these organs, as shown for *PsAO3* in *Pisum sativum* seeds [66]. Consequently, this elevated demand for Moco could only be fulfilled by an increased export rate of molybdate by MOT1.2. (ii) In general, developing seeds are loaded with a variety of important substances such as carbohydrates, storage proteins, oils and essential elements provided by the mother plant for the next generation [67]. Next to mineral macronutrients such as magnesium or sulfur, micronutrients such as iron or Mo are also transferred to seeds [68]. Especially for Mo (in the form of molybdate), no specific transport process for seed loading during maturation are currently known. The strong expression of *mot1.2* in fertilised ovaries observed in the present study as well as a reduced amount of molybdate in mature seeds of *mot1.2* KO plants observed by Gasber et al. [40] suggest that MOT1.2 might also be more actively involved in loading molybdate into developing seeds.

In summary, both MOT1 family members have important but different physiological roles in supplying the Moco biosynthesis complex with molybdate. MOT1.1 acts as a highly efficient absorber and distributor throughout the plant, whilst MOT1.2 provides access to stored molybdate for Moco biosynthesis. Nevertheless, there are still two missing links for molybdate distribution and the maintenance of its homeostasis: (i) How does molybdate enter leaf cells, and (ii) how is it imported into vacuoles? 

As MOT1 family members are not involved, a possible candidate to facilitate this task is the MOT2 family. The existence of this family has been reported for *A. thaliana* [48], whereas its function has not been further described [49].

## 4. Material and Methods

### 4.1. Plant Material

*Nicotiana benthamiana* wild type plants were used for the localisation, topology and protein–protein interaction studies. *Arabidopsis thaliana* (*A. thaliana*) wild type (ecotype *Col*-*0*) plants were used for stable transformation with endogenous *mo*t::*gfp-gus* constructs as well as being used as control plants. *A. thaliana* T-DNA insertion lines were ordered from NASC (Nottingham, UK) or were kindly provided by Professor Ekkehard Neuhaus (TU Kaiserslautern, Germany).

### 4.2. Cultivation of Plants in Soil

*N. benthamiana* was cultivated in common potting soil with a light/dark cycle of 10 h/14 h and temperatures between 22–25 °C under greenhouse conditions with artificial light (≈60 µE). Two weeks after germination, the seedlings were separated into 9 × 9 cm pots. The soil was mixed with NPK-fertiliser (1% Blaukorn^®^ classic Compo Expert, Münster Germany) and Perlite (5%). Plants were kept under sufficient water supply. Plants used for experiments were 5 to 12 weeks old. *A. thaliana* seeds were stratified at 4 °C for 48–72 h. Two weeks after germination, seedling plants were separated in 5 × 5 cm pots. Plants were cultivated in a walk-in phyto-chamber under a light/dark cycle of 10 h/14 h and temperatures between 22–25 °C, artificial light (≈60 µE) and 60–70% humidity.

### 4.3. Hydroponic Growth System

The hydroponic growth system was designed according to Conn et al. [55]. Germination and basal nutrient solutions (BNS: ¼ Hoagland’s solution) were modified in regard of their molybdate concentration to provide molybdate availability (+Mo, molybdate added to a concentration of 100 nM) and molybdate deficiency conditions (-Mo, molybdate was left out from the original ¼ Hoagland’s solution recipe). All BNS components were made with high-purity water (R = 18.2 MΩ) obtained from a Sartorius Arium Pro (Sartorius, Germany).

Seeds were individually sown to the lids of black microcentrifuge tubes containing 200 µL of germination solution that was solidified with 0.7% plant agar. Lids were transferred into germination tanks (AmphiRack, Biozym, Germany) that were filled with 250 mL of germination solution and, after a stratification phase (3 days at 4 °C in the dark) the plants were germinated and subsequently grown under short day conditions in a climate chamber (8 h light:16 h darkness, 60 µE, 22–25 °C, 60–70% humidity). The germination solution was gradually substituted with BNS over 3 days, and 8 days after sowing the lids of the germination tanks were removed and replaced with punctuated cling film to allow gas exchange. At the time of 20 days after sowing, microcentrifuge lids with the plants were transferred to lids with holes of the corresponding size of 50 mL centrifugation tubes cut off at the lower part, under the conical end and transferred to aerated tanks containing 8.5 L of basal nutrient solution with either +Mo or –Mo conditions. BNS was exchanged every 7–10 days. The plant material was harvested after 60 days.

From the same germination tanks, microcentrifuge lids with plants were also transferred to 130 mL centrifuge tubes with a hole in the lid of the corresponding size, 20 days after sowing. The plants were grown in an individual reservoir of 130 mL of BNS +Mo. BNS +Mo was exchanged every 7–10 days.

### 4.4. Leaf Area Determination and Phenotype Analysis

The determination of leaf area was conducted with Easy Leaf Area [69], a software that uses the colour ratio of each pixel of a digital photograph to discriminate between leaf and calibration area to estimate leaf area in a non-destructive and rapid manner. Leaf area determination was calculated after a germination phase of 6 days every 2 days over an overall time span of 20 days. The used calibration scale measured 2 × 2 cm. Photographs were taken with a Panasonic Lumix DMC-GX80 and a Panasonic H-FS 1442A camera lens (Panasonic, Kadoma, Japan). The software ran with auto-analysis settings and the mean was calculated from photographs taken at triplicates. In addition, a phenotyping analysis according to Boyés et al. [70] was performed on the same days. The growth stage of each plant was used to calculate the mean and standard deviation.

### 4.5. Molybdate Uptake Assay

Measurement of the molybdate concentration was conducted in a modified manner according to Cardenas and Mortensen [56]. The use of higher sample volumes made it possible to lower the detection limit of the assay to 10 nM of molybdate. In that way, the formed colour was concentrated, allowing a precise photometrical detection. The assay was performed with a sample volume of 50 mL stored in poly-propylene tubes. After adding 50 µL of sulfuric acid and 250 µL of assay reagent (2% (*w*/*v*) sodium hydroxide, 2 g/L 1,2-Dimercapto-4-methylbenzene, 16 mL/L thioglycolic acid) the samples were mixed thoroughly for 1 min and shook for 20 min. 1 mL of pure isoamyl acetate was added and the samples were mixed for 2 min and shook for 20 min. After an additional incubation placed on the lid, the forming organic phase was extracted from the conical end of the tube and transferred to a solvent-resistant cuvette. Extinction at 680 nm was measured and the molybdate concentration was determined using a calibration curve ranging from 0 to 150 nM of molybdate.

### 4.6. Nitrate Reductase Activity Assay

NR activity assay was carried out according to Dier et al. [71]. The leaf material of plants grown hydroponically was shock-frozen using liquid nitrogen and homogenized by a cooled mortar and pestle. To 100 mg of this material, 400 µL of extraction buffer [100 mM Hepes-KOH (pH 7.5); 5 mM magnesium acetate; 1 mM EDTA; 10% (*v*/*v*) glycerol; 0.5% (*w*/*v*) BSA; 0.1% Triton X-100; 1% polyvinylpolypyrrolidone (PVPP); 5 μM NaMoO_4_; 0.5 mM phenylmethylsulfonyl fluoride (PMSF); 5 mM dithiothreitol (DTT); 20 μM FAD; and 25 μM leupeptin] were added and mixed by several pipetting steps, followed by a subsequent centrifugation for 10 min at 4 °C at 21,000× *g* (Heraeus Fresco 21; Thermo Fisher, Waltham, MA, USA). A volume of 165 µL protein extract was mixed with pre-warmed (25.5 °C) assay buffer (100 mM Hepes-KOH; 6 mM KNO_3_; 0.6 mM NADH; 20 μM leupeptin; 12 μM FAD; 0.3 mM DTT; 6 μM Na_2_MoO_4_; 6 mM EDTA) and the NR reaction was stopped after 0, 10, 20 and 30 min, respectively, by removing 300 µL of reaction mixture and adding them to 25 µL of 600 mM zinc acetate. To remove unreacted NADPH, 75 µL 0.25 mM PMS solution was added to the samples followed by an incubation in the dark for 15 min. After addition of 300 µL each of 1% Sulfanilamid (SA in 3 M HCl) and 0.02% N-[Naphthyl-(1)]-ethylendiammoniumchloride (NED), samples were incubated for 35 min and formed NO_2_^−^ was determined colourimetrically at 540 nm. NO_2_^−^ concentration was quantified with a NO_2_^−^ standard curve. The resulting NR activity was given in nmol NO_2_^−^ per gram of fresh plant material per hour.

### 4.7. Cloning and Transformation of mot::gfp-gus Constructs

The tissue-specific expression pattern of *mot1.1* and *mot1.2* was analysed by cloning the promoter regions of each gene to express a GFP/GUS fusion protein in plants. Genomic regions of 1959 bp and 1962 bp, respectively, upstream of each start codon were amplified and subcloned into pDONR/Zeo via BP-reaction to generate entry vectors. Recombination of these entry vectors via LR reaction with pKGWFS7 [72] destination vectors resulted in expression vectors coding for a GFP-GUS fusion protein expressed under control of the endogenous *mot* promoters (*mot1.1*::*gus* and *mot1.2*::*gus*). Stable transformation of *A. thaliana* wild type plants was performed by floral dip according to Clough and Bent [73] using *Agrobacterium tumefaciens.* Selection for transformants of the T0 and T1 generation was carried out using kanamycin. 

### 4.8. Histochemical GUS-Assay

Transgenic *Arabidopsis* plants carrying the *mot::gus* constructs were grown hydroponically under molybdate availability conditions for 60 days. Positional effects of the integration locus of the constructs were avoided by use of several independently transformed lines. Plants were infiltrated with GUS-staining solution (9.6 mL 100 mM Tris/50 mM NaCl buffer pH 7.0, 0.2 mL 100 mM ferricyanide solution in Tris/NaCl buffer, 0.2 mL 100 mM X-Gluc solution in DMSO) according to Beeckman and Engler [74] using a vacuum chamber. Completely infiltrated tissue was incubated over night at 37 °C in the dark with a sufficient supply of oxygen. Subsequently, chlorophyll was extracted from the tissue using ethanol with concentrations of 30% (*v*/*v*), 60 % (*v*/*v*) and 96% (*v*/*v*) over a time span of 24 h each to allow a clear view of the coloured organs and tissue. Samples were kept in 96% (*v*/*v*) until photo documentation using a Keyence VHX digital microscope (Keyence, Osaka, Japan). Wild type control was free of background colouration (Figure S1).

### 4.9. Fluorimetric GUS-Assay

Plant material grown hydroponically for 60 days both under conditions of molybdate availability and deprivation, respectively, was separated into root, as well as into young and old leaves. A total of 100 mg of material was mixed with 500 µL GUS extraction buffer (0.1 M monosodium phosphate, 1 mM DTT) and homogenized using a Precellys 24 Homogeniser (Bertin Instruments, Montigny-le-Bretonneux, France) for two steps at 5500 rpm, each lasting 15 s. Subsequently, the samples were centrifuged for 20 min at 21,000× *g* and 4 °C, and 20 µL of the supernatant was loaded onto a 96-well plate in triplicates. After the addition of 100 µL prewarmed (37 °C) reaction buffer [50 mM Monosodium phosphate; 1 mM EDTA; 0.1% TritonX-100; 7 µL/mL ß-Mercaptoethanol; 0.33 mg/mL Methylumbelliferylglucuron (MUG); pH 7.0] fluorescence of the cleaved product MU (Methylumbelliferyl; excitation: 365 nm, emission: 455 nm) was measured every 2 min over a total time span of 40 min using a Tristar LB941 multimode reader (Berthold Technologies, Bad Wildbad, Germany). GUS activity was calculated from the gain of fluorescence over a time normalised to the amount of total protein and determined by Bradford assay using Roti^®^Quant reagent (Carl Roth, Karlsruhe, Germany). Root samples were measured with a dilution factor of 10.

### 4.10. Molecular Cloning and Transformation for Localisation, Topology/Co-Expression and Protein–Protein Interaction Studies

The coding sequences of *A. thaliana* molybdate transporters *mot1.1.* (AT2G25680) and *mot1.2* (AT1G80310) were amplified by PCR from *A. thaliana* cDNA using *attB* site-flanked primers to allow recombination of the respective PCR products into the pDONR/Zeo vector by BP reaction using the GATEWAY cloning system (Invitrogen, Waltham, MA, USA). For localisation studies, the resulting entry vectors were used for LR recombination into pDest-*GW-venus* and pDest-*venus-GW* vectors [20], thus generating expression vector coding for a fusion construct of the respective *mot* and the *venus* reporter. Cloning of a fusion construct harbouring Venus between N- and C-terminus of *mot1.1* was performed by the assembly of three single fragments each generated by a first PCR. Overlapping sequences were annealed during a second PCR and a full-length sequence was amplified. The *attB* site-flanked construct was subcloned into a pJET1.2 blunt vector (Thermo Scientific, USA) and then by BP reaction into the pDONR/Zeo vector. Recombination of the entry vector into pK7WG2 [72] using LR-reaction generated the expression vector pExp-*mot1.1N-venus-mot1.1C*. For co-localisation studies, fluorescent protein eqFP611 from sea anemone *Entacmaea quadricolor* served as a cytosolic marker [75].

For topology studies of MOT1.1 and MOT1.2 proteins, the Split-10+1-GFP system was used as a reporter [54]. GATEWAY compatible destination vectors were kindly provided by Professor Thordal-Christensen from the University of Copenhagen [54]. Expression vectors were cloned, enabling the fusion of GFP11 to either the N-or C-terminus of each MOT, respectively. The GFP1-10 fragment was either co-expressed cytosolically or combined with different cell organelle markers according to Nelson and colleagues [76]. Apoplastic localisation was achieved by using the signal peptide of *At*WAK2 (*A. thaliana* Wall Associated-Kinase 2 (AT1G21270); SP-GFP1-10) [77]. By adding the ER-retention signal peptide HDEL (His-Asp-Glu-Leu) to the C-terminus, ER-lumen localisation was achieved (SP-GFP1-10-HDEL) [78].

Fusion constructs required for interaction studies harbouring CDS of *mot1.1* and *mot1.2* were generated via subcloning into the pDONR/Zeo vector by BP reaction to create entry vectors. Plasma membrane localised *At*PIP2a (AT3G53420) [62] and tonoplast localised INT1 (AT2G43330) [63] served as negative controls. Cytosolic NSP3 (AT3G16390) [79] was used as an abundance control [28]. Fragments of CDS from these controls flanked by *attB* sites were generated by PCR and subcloned into pDONR/Zeo vector by BP reaction, resulting in entry vectors. Entry vectors were recombined into pDest-*scyce-GW*, pDest-*GW-scyce*, pDest-*vyne-GW* and pDest-*GW-vyne* [80] enabling their fusion to reporter fragments VYNE and SCYCE in all possible orientations regarding the N- and C-terminus of the resulting fusion protein. Generation of expression vectors containing CDS of *cnx1* in full-length (FL) as well as their E and G-domains in fusion with the reporter fragments VYNE and SCYCE in all possible orientations, is described by Kaufholdt et al. [20].

Transient expression in leaves of *N. benthamiana* necessary for localisation, topology and protein–protein interaction studies of *A. tumefaciens* harbouring the described expression vectors was realised by infiltration as described by Gehl et al. [80,81]. Protoplast isolation and the subsequent chemical transformation with generated expression vectors for localisation studies were carried out according to Negrutiu et al. [82]. Seedlings of *A. thaliana* were transiently transformed for localisation studies using the FAST (fast agro-mediated seedling transformation) method according to Li and colleagues [83].

### 4.11. Microscopy Detection

For all localisation and topology studies, as well as for interaction studies via BiFC, a confocal laser scanning microscope (cLSM) LSM 510 Meta from Zeiss (Göttingen, Germany) was used. Principles were described by Gehl et al. [80]. In brief, a cLSM-510META scanhead was connected to an Axiovert 200M. All specimens were examined either using a Plan-Neofluar 10×/0.3 or a C-Apochromat 40×/1.2 water-immersion objective. For excitation, both an argon laser (488 nm line for all VENUS approaches, as well as chlorophyll autofluorescence) or a Helium-Neon Laser (543 nm line for eqFP611) were used. Emitted light passed the primary beam-splitting mirrors UV/488/543/633 and was separated by a secondary beam splitter at 545 nm. Fluorescence was detected with filter sets as follows: BP 505–530 nm for all split-GFP (Em_max_: 510–515 nm) and VENUS (Em_max_: 525 nm) approaches; BP 560–615 for eqFP611 (Em_max_: 611 nm); LP 650 nm for chlorophyll autofluorescence. Bright field images were taken with a transmitted-light photomultiplier, and Lambda mode was used to examine the spectral signature of fluorophores. All images were taken using ZEISS Microscope Software ZEN 2009 and processed with ZEN lite and Fiji [84].

### 4.12. Bimolecular Fluorescence Complementation (BiFC) Protein–Protein Interaction Studies

BiFC studies were performed according to Gehl et al. [81] and Kaufholdt et al. [20]. The lower epidermis cells of 5–10 leaf discs from 2–3 *N. benthamiana* plants were analysed using identical cLSM settings to allow comparability. The interaction approach consisted of Cnx1 and the members of the MOT1 family fused to the reporter halves VYNE and SCYCE in different combinations and orientations. This was co-expressed in one leaf half. In the negative control, expressed in the second half of the leaf, MOT1.1 was replaced with PIP2a (the transporter in the outer membrane) and MOT1.2 was replaced with INT1 (the transporter in the tonoplast), respectively. Abundance controls were carried out in parallel BiFC studies in which the Cnx1 reporter fusion constructs were replaced with independent NSP3 reporter fusion constructs, allowing the estimation of different concentration levels of the negative control protein in correlation with the interaction approach counterpart [20].

## Figures and Tables

**Table 1:** Used KO plant lines with specification of AGI code, the literature and the source.

Gene	AGI Code	Literature	Source
*mot1.1*	AT2G25680	Tomatsu et al., 2007 [36]	NASC (SALK_118311)
*mot1.2*	AT1G80310	Gasber et al., 2011 [40]	NASC (SALK_015044C)
*mot1.1* and *mot1.2*	AT2G25680 and AT1G80310	Unpublished	Group of Ekkehard Neuhaus

## Data Availability

Not applicable.

## Supplementary Materials

The following supporting information can be downloaded at: https://www.mdpi.com/article/10.3390/molecules27103158/s1, Figure S1: Overview of histochemical GUS assay of different plant lines. Figure S2: Additional BiFC fusion orientations for MOT1.1/Cnx1 interaction studies. Figure S3: Additional BiFC fusion orientations for MOT1.2/Cnx1 interaction studies. Figure S4: Abundance controls for BiFC interaction studies. Table S1: List of used oligonucleotides. Table S2: List of used vectors.

## Sample Availability

Samples are available from the authors.

## Abbreviations

AO: aldehyde oxidase; BiFC: bimolecular fluorescence complementation; BNS: basic nutrient solution; Cnx: cofactor for nitrate reductase and xanthine dehydrogenase; cPMP: cyclic pyranopterin monophosphate; dKO: double knock-out; GFP: green fluorescent protein; GUS: β-glucoronidase; KO: knock out; Mo: molybdenum; Moco: molybdenum cofactor; Mo-enzymes: molybdenum dependent enzymes; MOT: molybdate transporter; MPT: molybdopterin; NR: nitrate reductase; SO: sulphite oxidase; SCYCE: C-terminus of the super cyan fluorescent protein; Venus: enhanced yellow fluorescent protein; VYNE: N-terminus of the enhanced yellow fluorescent protein; WT: wild type; XDH: xanthine dehydrogenase.
