# Supplementary material for: Physiological Importance of Molybdate Transporter Family 1 in Feeding the Molybdenum Cofactor Biosynthesis Pathway in Arabidopsis thaliana
Source: Molecules. 2022 May 15;27(10):3158. doi: 10.3390/molecules27103158 (PMC9147641; doi:10.3390/molecules27103158)
Supplement: Supplementary file 1 [file molecules-27-03158-s001.zip › molecules-1674487-supplementary.pdf]

Supplementary Materials

Physiological importance of molybdate transporter family 1 in feeding the molybdenum cofactor biosynthesis pathway in *Arabidopsis thaliana*

Rieke Minner-Meinen[1#], Jan-Niklas Weber[1#], Sarah Kistner[1], Paul Meyfarth[1], Merve Saudhof[1], Lena van den Hout[1], Jutta Schulze[1], Ralf-R. Mendel[1], Robert Hänsch[1,2]* and David Kaufholdt[1]

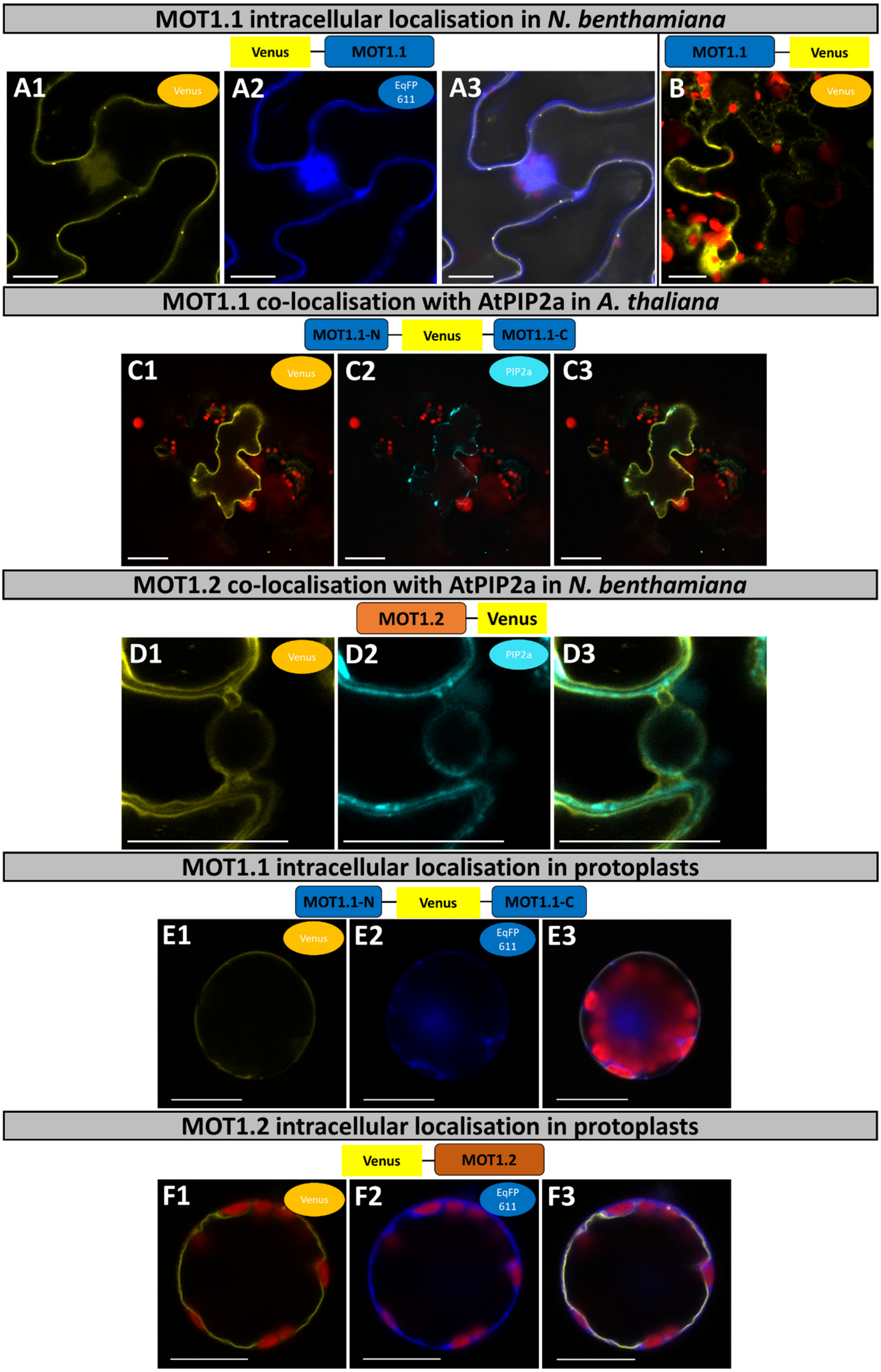

MOT1.1 intracellular localisation in N. benthamiana
Venus
MOT1.1
MOT1.1
Venus
A1
Venus
A2
EqFP 611
A3
B
Venus
MOT1.1 co-localisation with AtPIP2a in A. thaliana
MOT1.1-N
Venus
MOT1.1-C
C1
Venus
C2
PIP2a
C3
MOT1.2 co-localisation with AtPIP2a in N. benthamiana
MOT1.2
Venus
D1
Venus
D2
PIP2a
D3
MOT1.1 intracellular localisation in protoplasts
MOT1.1-N
Venus
MOT1.1-C
E1
Venus
E2
EqFP 611
E3
MOT1.2 intracellular localisation in protoplasts
Venus
MOT1.2
F1
Venus
F2
EqFP 611
F3

Figure S1: Additional microscopic pictures to the intracellular localisation of MOT1 family members. Studies with Venus fusion constructs were performed by transient transformation of (A/B/D) *N. benthamiana* and (C) *A. thaliana* via agrobacterium transformation as well as of (E/F) *N. benthamiana* mesophyll protoplasts via chemical transformation. Co-transformations were performed with (A/E/F) the cytosolic marker eqFP611 as well as with (C/D) the plasma membrane marker *At*PIP2a fused to SCFP. Shown are images from different channels: (A1/B/C1/D1/E1/F1) the Venus fluorescence channel, (A2/E2/F2) the eqFP611 fluorescence channel, (C2/D2) the SCFP fluorescence channel, A3/C3/D3/E3/F3) merged pictures. The chloroplast fluorescence channel (red) is merged in some cases. Images were taken 2-3 days after transformation. Each image was taken with a C-Apochromat 40x/1.2 water immersion objective and scale bars depict a length of 20 µm.

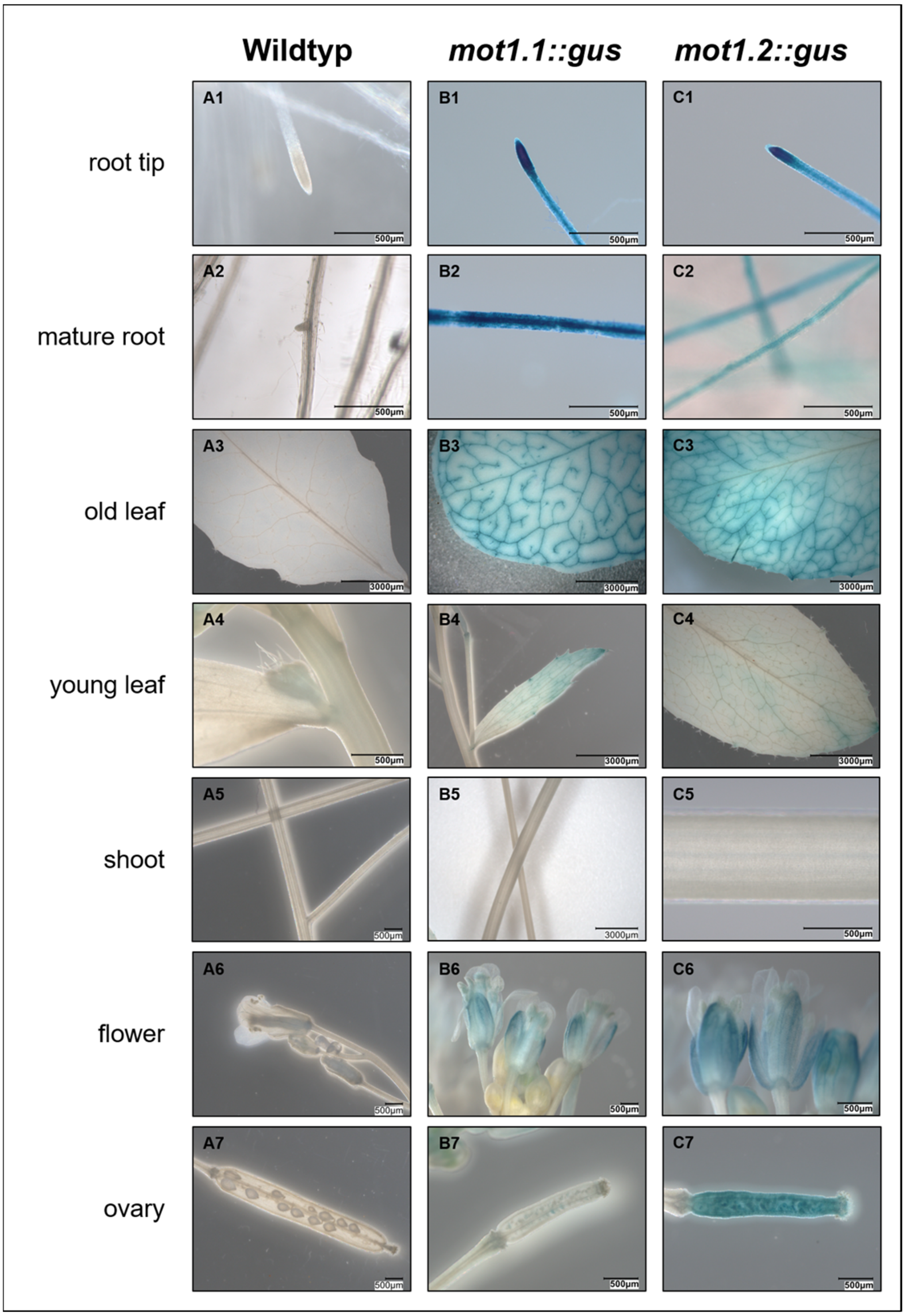

Figure S2: Overview of histochemical GUS assay of different plant lines. Depicted are different organs of WT (A), *mot1.1::gus* (B) and *mot1.2::gus* (C) lines: root tip (1), mature root (2), old leaf (3), young leaf (4), shoot (5), flower (6), ovary (7). The assay was performed with 60 days old plants grown hydroponically under molybdate availability conditions (BNS with 100 nM of molybdate). The scale bars are depicting a length of 500 µm and 3000 µm, respectively.

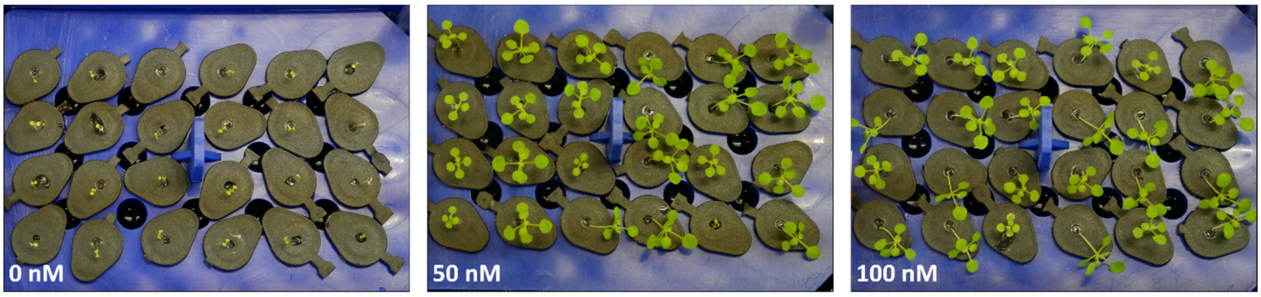

Figure S3: Overview of *Arabidopsis mot1.1 mot1.2* dKO grown for 20 days with 0 nM, 50 nM and 100 nM molybdate concentrations in hydroponics growth system.

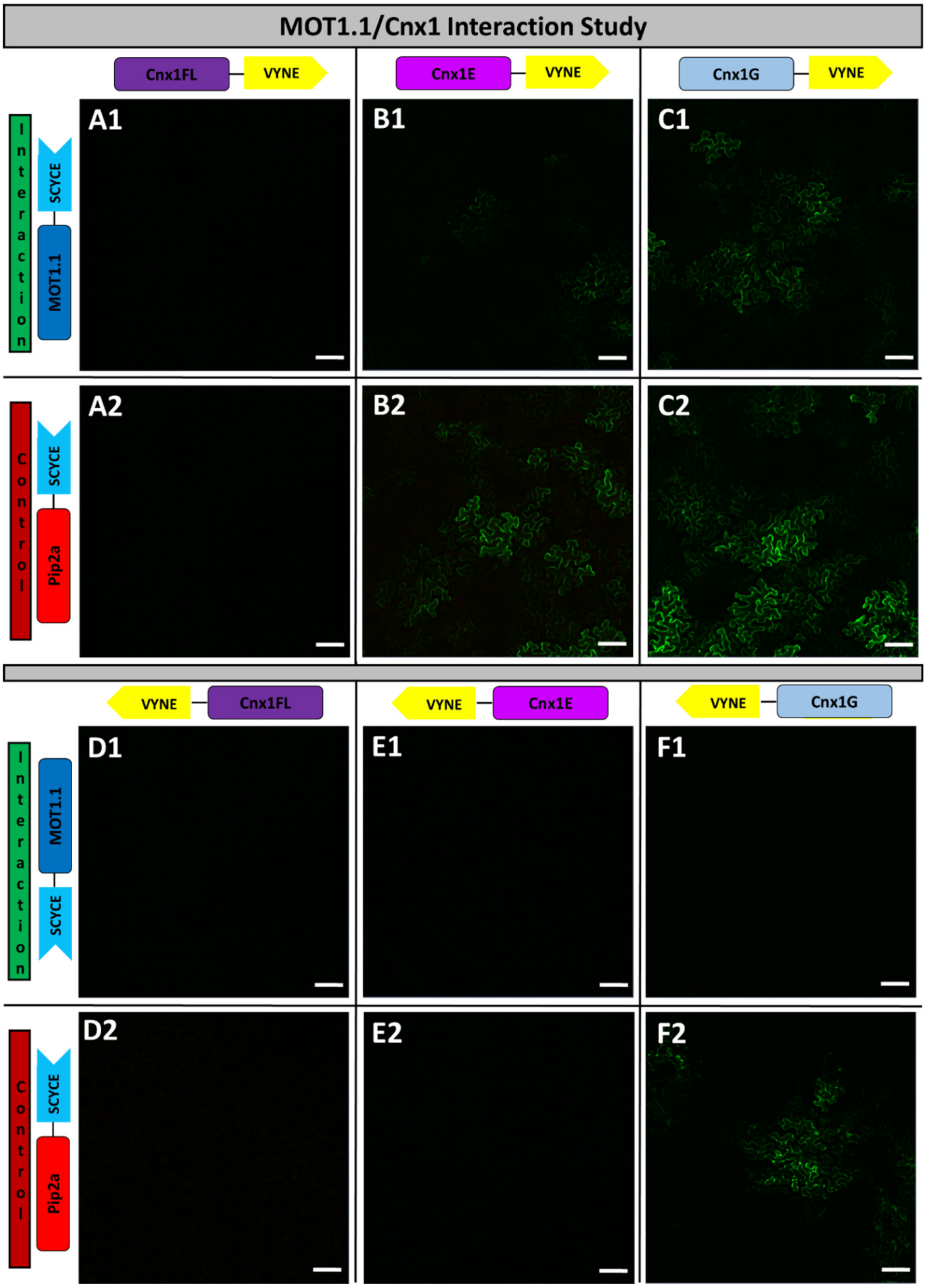

Figure S4: Additional BiFC fusion orientations for MOT1.1/Cnx1 interaction studies. Interaction approaches showing transiently transformed leaves of *N. benthamiana* co-expressing MOT1.1-SCYCE with Cnx1-VYNE (A1: Cnx1 full-length (FL)-VYNE, B1: Cnx1 E-domain (Cnx1E)-VYNE, Cnx1 G-domain (Cnx1G)-VYNE). In the negative controls (A2, B2, C2), PIP2a-SCYCE replaces MOT1.1-SCYCE. Analogous interaction approaches with SCYCE-MOT1.1 and VYNE-Cnx1FL (D1), VYNE-Cnx1E (E1) and VYNE-Cnx1G (F1) are shown. In the negative controls (D2, E2, F2), Pip2a-SCYCE replaces SCYCE-MOT1.1. Images were taken with a Plan-Neofluar 10x/0.3 and scale bars depict a length of 100 µm. Corresponding abundance controls are shown in Figure S4.

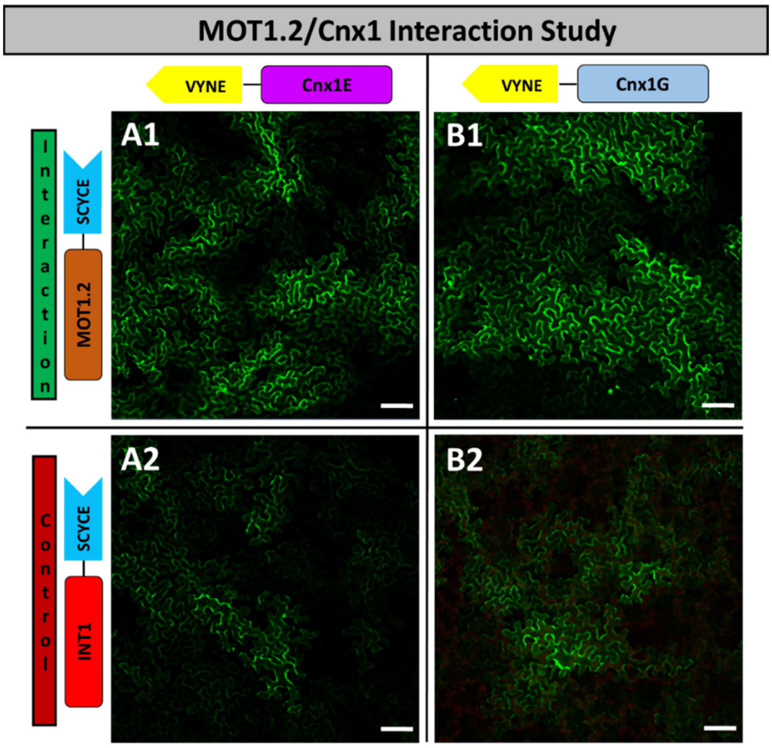

Figure S5: Additional BiFC fusion orientations for MOT1.2/Cnx1 interaction studies. Interaction approaches showing transiently transformed leaves of *N. benthamiana* co-expressing MOT1.2-SCYCE with VYNE-Cnx1E (A1) and VYNE Cnx1G (B1). In the negative controls (A1/B1), INT1-SCYCE replaces MOT1.2-SCYCE. Images were taken with a Plan-Neofluar 10x/0.3 and scale bars depict a length of 100 µm. Corresponding abundance controls are shown in Figure S4.

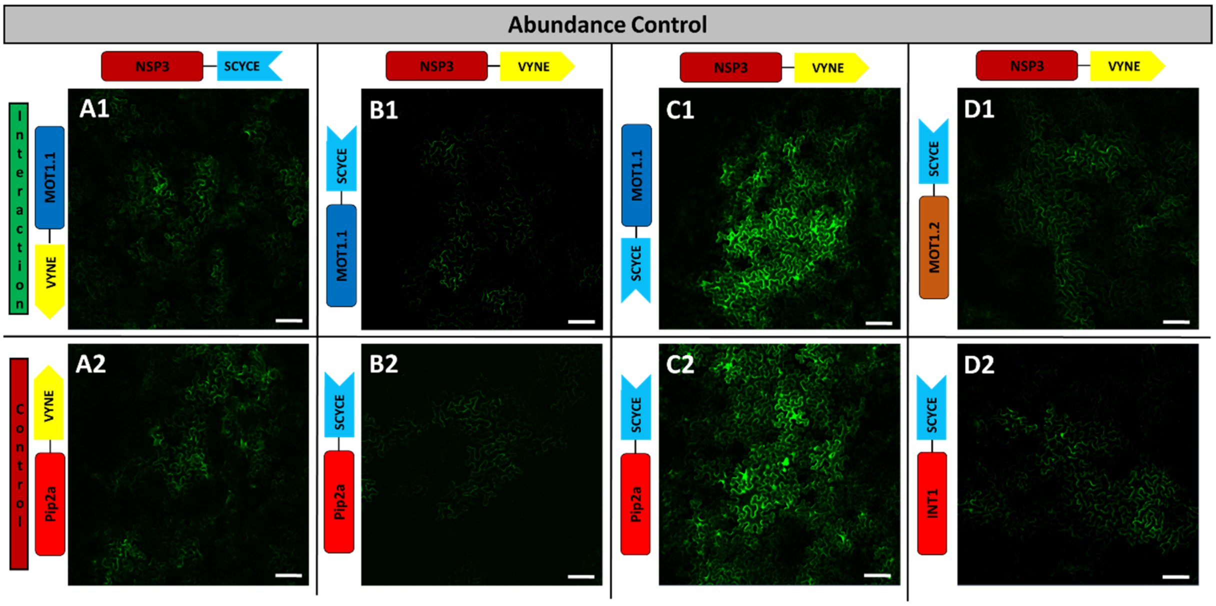

Figure S6: Abundance controls for BiFC interaction studies. Corresponding approaches of the transporters constructs co-expressed with the non-interacting construct NSP3 to evaluate the equal abundances of the used MOT-constructs and the negative controls. Images were taken with a Plan-Neofluar 10x/0.3 and scale bars depict a length of 100 µm.

Table S1: List of used oligonucleotides designed for genotyping, cloning of the endogenous *mot1.1* and *mot1.2* promoters, *mot1.1* and *mot1.2* CDS, plasma membrane and tonoplast markers.

No	Name	Sequence (5´-3´)	Purpose
1	*mot1.1*_geno_526-545_for	TGCCATGTCTGCGGTTAAGT	Genotyping *mot1.1* locus
2	*mot1.1*_geno_979-998_rev	ATGCAGCCGAGAACTCCTTC	Genotyping *mot1.1* locus
3	pROK2_101-121_rev	AATCAGCTGTTGCCCGTCTC	Genotyping pROK2 insert
4	*mot1.2*_geno_1160-1179_for	CGGCGATTCCTCAGATTCCA	Genotyping *mot1.2* locus
5	*mot1.2*_geno_1682-1701_rev	ATCGACCTGCGACTCATCAC	Genotyping *mot1.2* locus
6	*mot1.1*_(-1959) _attB1_for	GGGGACAAGTTTGTACAAAAAAGCAGGCTTA ATATCGTGTGTATTTACTACAAGC	Cloning of endogenous *mot1.1* promoter
7	*mot1.1*endo-attB2-rev	GGGGACCACTTTGTACAAGAAAGCTGGGTCT GTTTCTGTTTTGTGGTTTGTATC	Cloning of endogenous *mot1.1* promoter
8	*mot1.2*_(-1962) _attB1_for	GGGGACAAGTTTGTACAAAAAAGCAGGCTTA GACTTGGAGCCAATGTTGCAC	Cloning of endogenous *mot1.2* promoter
9	*mot1.2*endo-attB2-rev	GGGGACCACTTTGTACAAGAAAGCTGGGTCG ATTGGATCTAAAGTCAAAGCTC	Cloning of endogenous *mot1.2* promoter
10	*mot1.1*endo_seq1_for	GTGCAAATTGAGCTTCCATAGC	Sequencing of endogenous *mot1.1* promoter
11	*mot1.2*endo_seq1_for	GACTTGGAGCCAATGTTGCAC	Sequencing of endogenous *mot1.2* promoter
12	*mot1.1*_attB1-for	GGGGACAAGTTTGTACAAAAAAGCAGGCTTA ACCATGGAGTCTCAGTCTCAGAGAGG	Cloning of *mot1.1* CDS
13	*mot1.1*_attB2-rev_stop	GGGGACCACTTTGTACAAGAAAGCTGGGTCT CAAGCATGTTCACCGGATTGCG	Cloning of *mot1.1* CDS
14	*mot1.1*_attB2-rev_nostop	GGGGACCACTTTGTACAAGAAAGCTGGGTCA GCATGTTCACCGGATTGCGGG	Cloning of *mot1.1* CDS
15	*mot1.1n-venus*_fus_for	G AAGAGGAAGA GGAAGAAGGA ATGGTGAGCAAGGGCGAGGAGCTG	Cloning of *mot1.1n-venus-mot1.1c* fusion construct
16	*mot1.1n-venus*_fus_rev	CAGCTCCTCGCCCTTGCTCACCATTCCTTCTTCC TCTTCCTCTTC	Cloning of *mot1.1n-venus-mot1.1c* construct

17	*venus-mot1.1c*_fus_for	CTCGGCATGGACGAGCTGTACAAG GATGGTTCGA GAGGAAGAGG	Cloning of *mot1.1n-venus-mot1.1c* construct
18	*venus-mot1.1c*_fus_rev	CCTCTTCCTCTCGAACCATCCTTGTACAGCTCG TCCATGCCGAG	Cloning of *mot1.1n-venus-mot1.1c* construct
19	*mot1.2*_attB1-for	GGGGACAAGTTTGTACAAAAAAGCAGGCTTA ACCATGGAGACAACTACAACTCCTCTGC	Cloning of *mot1.2* CDS
20	*mot1.2*_attB2-rev_stop	GGGGACCACTTTGTACAAGAAAGCTGGGTCTT AGACATCACGAGGAGCGGCTTCG	Cloning of *mot1.2* CDS
21	*mot1.2*_attB2-rev_nostop	GGGGACCACTTTGTACAAGAAAGCTGGGTCG ACATCACGAGGAGCGGCTTCG	Cloning of *mot1.2* CDS
22	AttB1-*pip2a*_for	GGGGACAAGTTTGTACAAAAAAGCAGGCTTA ACCATGGCAAAGGATGTGGAAGCCG	Cloning of plasma membrane marker
23	AttB2_pip2a_nostop_rev	GGGGACCACTTTGTACAAGAAAGCTGGGTCG ACGTTGGCAGCACTTCTGAATG	Cloning of plasma membrane marker
24	AttB1_*int1*_for	GGGGACAAGTTTGTACAAAAAAGCAGGCTTA ACC ATGACATTGACGATCCCAAA	Cloning of tonoplast marker
25	AttB2_*int1*_stop_rev	GGGGACCACTTTGTACAAGAAAGCTGGGTC TCA AGATTGAGATCCCTGCTCGAG	Cloning of tonoplast marker
26	AttB2_ *int1*_nostop_rev	GGGGACCACTTTGTACAAGAAAGCTGGGTC AGATTGAGATCCCTGCTCGAG	Cloning of tonoplast marker

Table S2: List of used Plasmids for localization and topology studies, generation of transgenic *mot::gus* plant lines and protein-protein interaction studies. Zeo = zeocin, Kan = kanamycin, Cmp = chloramphenicol, Spec = spectino-mycin.

No.	Name	Reporter	Orientation	Resistance Bacteria / Plant	Source
1	pDonor-Zeo			Zeo/ -	
2	pEntry-*mot1.1*-stop			Zeo / -	
3	pEntry-*mot1.1*-nostop			Zeo / -	
4	pEntry-*mot1.1*N-*venus*-*mot1.1*C			Zeo / -	
5	pEntry-*mot1.2*-stop			Zeo / -	
6	pEntry-*mot1.2*-nostop			Zeo / -	
7	pDest-*venus*-GW	Venus	N-terminus	Kan & Cmp / -	Kaufholdt *et al*., 2013
8	pDest-GW-*venus*	Venus	C-terminus	Kan & Cmp / -	Kaufholdt *et al*., 2013
9	pExp-*mot1.1-venus*	Venus	C-terminus	Kan / -	
10	pExp-*venus-mot1.1*	Venus	N-terminus	Kan / -	
11	pExp-*mot1.1N-venus-mot1.1C*	Venus		Spec / -	
12	pExp-*mot1.2-venus*	Venus	C-terminus	Kan / -	
13	pExp-*venus-mot1.2*	Venus	N-terminus	Kan / -	
14	pExp-*eqfp611*	eqFP611		Spec / -	Gehl *et al*., 2011
15	pDest-*gfp11*-GW	GFP11	N-terminus	Spec & Cmp / -	Xie *et al., 2017*
16	pDest-GW-*gfp11*	GFP11	C-terminus	Spec & Cmp / -	Xie *et al., 2017*
17	pExp-*mot1.1-gfp11*	GFP11	C-terminus	Spec / -	
18	pExp-*gfp11-mot1.1*	GFP11	N-terminus	Spec / -	
19	pExp-*mot1.2-gfp11*	GFP11	C-terminus	Spec / -	
20	pExp-*gfp11-mot1.2*	GFP11	N-terminus	Spec / -	
21	pExp-*gfp1-10*	GFP1-10		Spec / -	
22	pExp-*SP-gfp1-10*	GFP1-10		Spec / -	
23	pExp-*SP-gfp1-10-HDEL*	GFP1-10		Spec / -	
24	pEntry-pEndo*mot1.1*			Zeo / -	

No.	Name	Reporter	Orientation	Resistance Bacteria / Plant	Source
25	pEntry-pEndo*mot1.2*			Zeo / -	
26	pDest-*GW-gfp-gus* (pKGWFS7)	GFP-GUS		Spec & Cmp	Karimi *et al.*, 2002
27	pExp-*mot1.1::gfp-gus*	GFP-GUS		Spec / Kan	
28	pExp-*mot1.2*::*gfp-gus*	GFP-GUS		Spec / Kan	
29	pEntry-*Atpip2a*-nostop			Zeo / -	
30	pEntry-*int1*-nostop			Zeo / -	
31	pDest-*scyce*-GW	SCYCE	N-terminus	Kan & Cmp	Gehl *et al.*, 2009
32	pDest-GW-*scyce*	SCYCE	C-terminus	Kan & Cmp	Gehl *et al.*, 2009
33	pDest-*vyne*-GW	VYNE	N-terminus	Kan & Cmp	Gehl *et al.*, 2009
34	pDest-GW-*vyne*	VYNE	C-terminus	Kan & Cmp	Gehl *et al.*, 2009
35	pExp-*scyce-cnx1FL*	SCYCE	N-terminus	Kan / -	Kaufholdt *et al.*, 2013
36	pExp-*cnx1FL-scyce*	SCYCE	C-terminus	Kan / -	Kaufholdt *et al.*, 2013
37	pExp-*vyne-cnx1FL*	VYNE	N-terminus	Kan / -	Kaufholdt *et al.*, 2013
38	pExp-*cnx1FL-vyne*	VYNE	C-terminus	Kan / -	Kaufholdt *et al.*, 2013
39	pExp-*scyce-cnx1E*	SCYCE	N-terminus	Kan / -	Kaufholdt *et al.*, 2013
40	pExp-*cnx1E-scyce*	SCYCE	C-terminus	Kan / -	Kaufholdt *et al.*, 2013
41	pExp-*vyne-cnx1E*	VYNE	N-terminus	Kan / -	Kaufholdt *et al.*, 2013
42	pExp-*cnx1E-vyne*	VYNE	C-terminus	Kan / -	Kaufholdt *et al.*, 2013
43	pExp-*scyce-cnx1G*	SCYCE	N-terminus	Kan / -	Kaufholdt *et al.*, 2013
44	pExp-*cnx1G-scyce*	SCYCE	C-terminus	Kan / -	Kaufholdt *et al.*, 2013
45	pExp-*vyne-cnx1G*	VYNE	N-terminus	Kan / -	Kaufholdt *et al.*, 2013
46	pExp-*cnx1G-vyne*	VYNE	C-terminus	Kan / -	Kaufholdt *et al.*, 2013
47	pExp-*scyce-mot1.1*	SCYCE	N-terminus	Kan / -	
48	pExp-*vyne-mot1.1*	VYNE	N-terminus	Kan / -	
49	pExp-*scyce-mot1.2*	SCYCE	N-terminus	Kan / -	
50	pExp-*mot1.2-scyce*	SCYCE	C-terminus	Kan / -	
51	pExp-*vyne-mot1.2*	VYNE	N-terminus	Kan / -	

No.	Name	Reporter	Orientation	Resistance Bacteria / Plant	Source
52	pExp-*mot1.2-vyne*	VYNE	C-terminus	Kan / -	
53	pExp-*Atpip2a-vyne*	VYNE	C-terminus	Kan / -	
54	pExp-*int1-scyce*	SCYCE	C-terminus	Kan / -	
55	pExp-*nsp3-scyce*	SCYCE	C-terminus	Kan / -	Kaufholdt *et al.*, 2013
56	pExp-*nsp3-vyne*	VYNE	C-terminus	Kan / -	Kaufholdt *et al.*, 2013
